# Supplementary material for: Cheminformatics identification of modulators of key carbohydrate-metabolizing enzymes from C. cujete for type-2 diabetes mellitus intervention
Source: J Diabetes Metab Disord. 2023 Jul 1;22(2):1299–317. doi: 10.1007/s40200-023-01249-7 (PMC10638353; doi:10.1007/s40200-023-01249-7)
Supplement: Supplementary file 1 — Supplementary Material 1 [file 40200_2023_1249_MOESM1_ESM.docx]

**Cheminformatics identification of modulators of key carbohydrate-metabolizing enzymes from *C. cujete* for type-2 diabetes mellitus intervention**

Fatai Oladunni Balogun^1^, Karishma Singh^1,2^, Athika Rampadarath^1^, Ayesha Akoonjee^1^, Kayleen Naidoo^1^, Saheed Sabiu^1^*

^1^Department of Biotechnology and Food Science, Faculty of Applied Sciences, Durban University of Technology, P.O. Box 1334, Durban, 4000, South Africa.

^2^Department of Nature Conservation, Mangosuthu University of Technology, South Africa

*Correspondence: sabius@dut.ac.za

**Abstract**

**Purpose**: The therapeutic use of oral hypoglycaemic agents in the management of type-2 diabetes mellitus (T2DM) is without adverse effects; thus, calls for alternative and novel candidates from natural products in medicinal plants.

**Method**: The study explored molecular docking and molecular dynamics (MD) simulation approaches to identify key antidiabetic metabolites from *Crescentia cujete*.

**Results**: Molecular docking results identified four and/or five best compounds against each target enzyme (alpha-glucosidase, dipeptidyl peptidase-IV, aldose reductase, and protein tyrosine phosphatase-1B (PTP-1B)) implicated in diabetes. The resulting complexes (except against PTP-1B) had higher docking scores above respective standards (acarbose, Diprotin A, ranirestat). The MD simulation results revealed compounds such as benzoic acid (-48.414 kcal/mol) and phytol (-45.112 kcal/mol) as well as chlorogenic acid (-42.978 kcal/mol) and naringenin (-31.292 kcal/mol) had higher binding affinities than the standards [acarbose (-28.248 kcal/mol), ranirestat (-21.042 kcal/mol)] against alpha-glucosidase and aldose reductase, respectively while Diprotin A (-45.112 kcal/mol) and ursolic acid (-18.740 kcal/mol) presented superior binding affinities than the compounds [luteolin (-41.957 kcal/mol and naringenin (-16.518 kcal/mol)] against DPP-IV and PTP-1B respectively.

**Conclusion**: While isoflavone (alpha-glucosidase), xylocaine (DPP-IV), luteolin (aldose reductase,) and chlorogenic acid (PTP-1B) were affirmed as the best inhibitors of respective enzyme targets, luteolin, and chlorogenic acid may be suggested and proposed as probable candidates against T2DM and related retinopathy complication based on their structural stability, compactness and affinity for three (DPP-IV, aldose reductase, and PTP-1B) of the four targets investigated. Further studies are warranted *in vitro* and *in vivo* on the antihyperglycaemic effects of these drug candidates.

**Keywords**: Chlorogenic acid, *Crescentia cujete*, Isoflavone, Luteolin, Molecular dynamics simulation, Type-2 diabetes mellitus, Xylocaine

**Introduction**

Diabetes mellitus (DM) is an endocrine illness with increasing prevalence globally, thus, constituting a continuous health challenge with resulting huge financial implications. Diabetes mellitus is majorly classified into type-1 DM (i.e., insulin-dependent) and type-2 DM (non-insulin-dependent) with the latter accounting for 90-95% of DM incidence [1-2]. The disease (type-2 diabetes mellitus) is reported to be attributed to a hyped concentration of glucose (hyperglycaemia) and insulin (hyperinsulinemia) within the systemic circulation [3] arising from the inability of beta cells of the islets of Langerhans within the pancreas to secret insulin or the body tissues not responding to the available insulin [4]. The prevalence of the menace on a yearly basis has continued to remain an issue of concern for researchers (particularly diabetic), healthcare practitioners, etc., globally. In fact, according to the International Diabetes Foundation, 425 million patients are affected globally by T2DM in 2017 [5] with a further increase to 463 million in 2019 [2] and an anticipated increase to 764 million by the year 2030 [6], if no suitable solution is found.

In the past, various efforts in the management of the T2DM are tailored toward maintaining the glucose at a normal level via non-pharmacological interventions such as diets and exercise and pharmacologically using oral hypoglycaemic agents (OHAs) including the likes of sulphonylureas, biguanides, meglitinides, thiazolidinediones and alpha-glucosidase inhibitors. Thus, preventing its likely complications including retinopathy, neuropathy, and nephropathy among others [7]. The individual members or classes of these drugs (OHAs) are sometimes able to regulate the elevated glucose levels to optimal level via various mechanisms and/ or involving the inhibition of specific protein targets such as alpha-glucosidase, dipeptidyl peptidase -IV (DPP-IV), protein tyrosine phosphatase-1 B (PTP-1B) implicated in the emergence of T2DM and aldose reductase as a consequent of diabetes retinopathy.

Alpha-glucosidase is a carbohydrate-hydrolyzing enzyme of the intestines involved in the continuous breakdown of disaccharides (cleaving α-1,4-glycopyranosidic linkage) to produce simple sugars such as glucose [8-9] during the postprandial hyperglycaemic state. While dipeptidyl peptidase IV (DPP-IV) is a surface antigen protein involved in the cleavage of the glucagon peptide-like 1 (GLP-1) and glucose-dependent insulinotropic peptide (GIP) involved in the potentiation of pancreatic cells to secrete insulin [10-11], PTP-1B catalyzes the dephosphorylation of insulin receptors (IR) and insulin receptor substrates (IRS) concerned with type-2 diabetes mellitus (T2DM) pathogenesis and obesity inducing insulin and leptin resistance, respectively [12].

Diabetes retinopathy (DR) is one of the prominent microvascular complications of diabetes [13] and a major cause of blindness in working-class individuals globally [14]. While more than 60% of T2DM cases have been reported to progress into DR after few years [15], in terms of prevalence, more than 100 million individuals suffer from this ever-increasing health challenge worldwide [14]. Aldose reductase is a key enzyme involved in polyol pathway and it is regarded as a clinical therapeutic target for complications arising from DR [16].

The inhibition of these carbohydrate enzymes by various classes of drugs (acarbose, Diprotin A, ursolic acid, ranirestat, etc.) is considered a good therapeutic approach toward diabetes control though these OHAs are recognized with side effects including flatulence, stomachache, and diarrhoea [9]. Due to the prevalence and severity of T2DM including its complications and coupled with emanating side effects from available synthetic inhibitors, the search for novel and natural compounds with antidiabetic activity is seen as a viable method or intervention if it is to be well managed [17-19]

Medicinal plants use for the treatment of numerous diseases have been an age-long exploration or tradition [20-21]. In fact, at a time when there has been non-existence in the use of chemical or synthetic moieties, humankind have continued to relied on these natural products or medicinal plant formulations to take care of illnesses [22] that may compromise the state of their health or threatens their survival as a race. While these medicinal plants, not limited to *Crescentia cujete* and others are (naturally) available, relatively cheap and with adjudged little or no side effects [23], they are established with one or more biological and/ or pharmacological attributes owing to the inherent compounds, many of which have in recent times been developed into drug candidates against a number of ailments [18-19, 24].

*Crescentia cujete* L., commonly known as calabash tree is an important indigenous tree with wide medicinal uses including diabetes, hypertension, cancer, infectious illnesses, infertility problems attributed to a number of verified pharmacological and biological activities such as antidiabetic, antibacterial, anti-inflammatory, anthelmintic, antioxidant, antivenom, cytotoxic, neuroprotective etc., [24]. The plant belonging to the family Bignoniaceae [25] is also endowed with good nutritional potential and economic benefit. The recently published critical review of the plant provided insights into several classes of phytocompounds including flavonoids, phenolic acids, alkaloids, saponins, tannins etc. from different parts of the plant responsible for various established pharmacological potentials [24]. Inclusively, the antidiabetic property of the various parts (leaves, stem bark and roots) of the plant in various extracts (aqueous, hydroethanol, ethanol) have been investigated in both *in vitro* and *in vivo* models [26-30]. Ethanol extract and fractions (hexane, diethyl ether and ethyl acetate) in a study by Adeyemi et al. [29] showed comparative inhibition with acarbose against alpha-amylase with a superior effect over the standard against alpha-glucosidase. Additionally, aqueous root extract at 250 mg/kg body weight (b.w.) caused a reduction in the increased glucose level (brought about by alloxan) of diabetic Sprague-Dawley rats from 302.75 to 119.00 mg/dl, an effect comparable with glibenclamide (5 mg/kg b.w.) [26]. Similarly, aqueous leaves extract at concentrations of 200 and 400 mg/kg b.w. was reported to reverse elevated glucose level of streptozotocin -induced hyperglycaemic Wistar rats [28]. Despite these reports, the molecular mechanism of antidiabetic action of the plant remains elusive. The present study is aimed at studying the interaction of natural products (secondary metabolites) in *Crescentia cujete* L with key targets implicated in T2DM through computational studies as a way of discovering novel therapeutic agents with antidiabetic properties.

**Materials and Methods**

*Data mining for C. cujete metabolites*

The identification of the 56 phytoconstituents (Supplementary Table S1) of *C. cujete* provided in the work of Balogun and Sabiu [24] was confirmed using various databases [NPASS (http://bidd.group/NPASS/) and Dr. Duke’s Phytochemical and Ethnobotanical Database (https://phytochem.nal.usda.gov/phytochem/search)].

*Targets, molecular docking, and molecular dynamic simulation*

The crystal structure for the target enzymes; PDB: 3W37 (alpha-glucosidase); PDB: 1IEI (aldose reductase); PDB: 1WCY (dipeptidyl-peptidase 4) and PDB: 2HNQ (protein tyrosine phosphatase 1B)] was obtained from the RSCB Protein Data Bank (https://www.rcsb.org/) and downloaded in PDB format. They are prepared on UCFS chimera software v. 1.14 and saved in PDB format based on [31] method. The 3D structures of the compounds and reference standards (acarbose, ranirestat, Diprotin A, ursolic acid) were collected from PubChem (https://pubchem.ncbi.nlm.nih.gov/) in sdf format, thereafter, prepared and optimized using Avogadro software and then saved as mol2 according to Sabiu et al. [19].

The molecular docking of the optimized ligands (*C. cujete* metabolites and reference standards) and proteins (alpha-glucosidase, aldose reductase, DPP-IV and PTP-1B) was performed using Python Prescription (PyRx) version 0.9.6. as described by Ambrose et al. [32]. Briefly, following the description in the work of Mubarak et al. [33], the x-y-z coordinates of the active sites of the proteins were identified where the docking of the ligands occurred, thereafter, the grid box was adjusted to fit perfectly within the well-defined x-y-z coordinates. AutoDock Vina (version 4.2)-coupled PyRx tools was employed to implement the molecular docking of the optimized ligands and prepared proteins. The ligands were then ranked based on the best affinities for the studied proteins (alpha-glucosidase, aldose reductase, dipeptidyl-peptidase IV, and protein tyrosine phosphatase-1B) and the highest affinity of complexes with the best pose formed. Consequently, the top compounds with the most negative binding scores were selected as the best compounds against each enzyme and the inhibition constant (Ki) of their resulting complexes was obtained from their binding energy (**Δ**G) [34], before further evaluation through MD simulation.

The MD simulation was executed using the AMBER 18 software (Centre for High-Performance Computing) as earlier described [19], where the FF18SB variant of the amber force field was employed to provide the operating system functions. Using the ANTECHAMBER, the atomic partial charges of the ligand via general amber force fields (GAFF) and restrained electrostatic potentials (RESP) were created. The TIP3P potential represented water molecules, Na^+^ and Cl ^-^ counter ions were added to neutralize the system and the cut-off value of the non-bond interactions was set to 8 Å. The total simulation was carried out for 100 ns, with the Leap module SHAKE algorithm adopted to limit the expansion of chemical bonds involving hydrogen atoms. For each individual simulation was a 2 fs step size corresponding to an isobaric-isothermal ensemble (NPT) containing randomized seeding, 300 K temperature, 1 bar constant pressure, Langevin thermostat (1.0 ps collision frequency), and 2 ps pressure-coupling constant. Examined MD simulation results were presented as post-dynamic data.

The post-dynamic simulation was achieved as previously described [19]. The AMBER 14 PTRAJ program was used to combine and analyze the coordinates of the systems, followed by CPPTRAJ program used for the analyses of parameters such as root mean square fluctuation (RMSF), root mean square deviation (RMSD), radius of gyration (Rg), and solvent accessible surface area (SASA). Molecular Mechanics/GB Surface Area method (MM/GBSA) was employed to calculate the average binding free energy for each ligand-protein complex over 100 000 snapshots from the 100 ns simulation. Discovery Studio version 21.1.0 was used to analyze and visualize the interaction of the complexes for each interaction. All the data plots were generated using Origin data analysis software V18 [35], and where applicable, statistical significance between calculated means was considered at p < 0.05 level.

*Pharmacokinetics evaluation*

The pharmacokinetic profiles including absorption, distribution, metabolism and excretion (ADME) and drug-likeness properties of the compounds with the most promising complex were predicted with the SWISS ADME (Swiss Institute of Bioinformatics, Lausanne, Switzerland) server (http://swissadme.ch/index.php).

**Results and discussions**

The global scientific databases are filled with large repertoire of probable compounds identified from medicinal plants or natural products that are continuously being submitted or reported from published studies on timely basis. It is of interest to note that researchers are often times comfortable to pick alien or new medicinal plant species to study in an effort to identify their compounds but many a time they lacked the tenacity to further explore or screen these (literature available) avalanches of compounds for likely drug development. It is on this aforementioned background that prompted the development of this theoretical approach (through computational tool) with a view at making or identifying possible candidates of pharmacological importance (against a number of diseases) from the plant. Computational analyses are extensively considered during drug discovery and development to screen libraries of compounds with the potential to elicit the desired therapeutic activity [36]. Computational methods allow for the elimination of compounds without therapeutic benefit prior to further experimental and/or clinical studies such as *in vitro* and *in vivo* testing.

*Molecular docking*

The docking scores obtained from the interaction of the 56 *C. cujete* compounds with the respective active sites of the four investigated targets are presented in Supplementary Table S1, where most of the compounds that exhibited, lower negative scores were compared to the reference standards. However, the findings of the selected top compounds (based on best binding scores) against each target are presented in Table 1. Compounds such as isoflavone (-7.7 kcal/mol), luteolin (-6.7 kcal/mol), and naringenin (-9.9 kcal/mol) with specific targets (alpha-glucosidase, DPP-IV, aldose reductase, respectively) revealed the most negative binding scores when compared with complexes for the respective standards [acarbose (-5.0 kcal/mol), Diprotin A (-5.5 kcal/mol), ranirestat (-9.0 kcal/mol). Chlorogenic acid with the best binding score (-6.2 kcal/mol) against PTP-1B had a lower binding score compared to the standard, ursolic acid (-7.4 kcal/mol) (Table 1). Molecular docking analysis measures the binding mode of an inhibitor or compound as it binds to a protein [37]. Molecular docking uses a scoring function to determine the inhibitor with the most appropriate (binding) orientation at the active site of an enzyme. The most negative binding score depicts the better pose and the best modulator of the enzyme [19]. The highest negative binding scores of isoflavone, luteolin, naringenin, and chlorogenic acid observed in this study were an indication of their superior affinities for the respective (alpha-glucosidase, DPP-IV, aldose reductase) targets above other compounds and standards except for ursolic acids against PTP-1B. The superior binding mode of phytocompounds over their synthetic counterpart against these targets has been reported [19]. Typically, compounds such as luteolin 7-O-beta-D-glucoside (-8.6 and -9.4 kcal/mol), 1,3-dicaffeoylquinic acid (-8.1, -9.7 kcal/mol), epicatechin (-7.7, -9.6 kcal/mol) etc. from *Carpobrotus edulis* had enhanced binding scores superior to acarbose and ranirestat (-7.5 and -8.4 kcal/mol respectively) against alpha-glucosidase (PDB: 3W37) and aldose reductase (PDB: 3RX3) [19]. Additionally, 3-caffeoylquinic acid (-7.8 kcal/mol), engeletin (-8.4 kcal/mol, and sinocrassosideA1 (-9.0 kcal/mol) from *Helichrysum petiolar* had higher binding affinity values than acarbose (-6.3 kcal/mol) against alpha-glucosidase (PDB: 3WEL) [38]. Thus, our findings in this study corroborated earlier studies [19, 39], on the best pose of some phytoconstituents as good modulators of the studied enzymes.

Inhibition constant (Ki) aimed to measure the strength of binding occurring between the inhibitor and the enzyme [40-41]. A lower Ki value is reported as an indication of a stronger affinity between the inhibitor and the enzyme [36]. As observed from the study, the Ki of these compounds, i.e., isoflavone, luteolin and naringenin complexed with respective enzymes (alpha-glucosidase, DPP-IV and aldose reductase) were the lowest (0.05, 0.07 and 0.02 µM, respectively) compared to the standards [acarbose (0.14 µM), Diprotin A (0.12 µM) and ranirestat (0.03 µM). However, the complex formed between ursolic acid (standard) and PTP-1B revealed a lower Ki (0.06 µM) compared with complexes of the phytocompounds with the enzyme (Table 1). The lower Ki values of isoflavone, luteolin and naringenin compared to the standards signify their stronger and superior inhibitory effects on the respective enzymes (alpha-glucosidase, DPP-IV, aldose reductase). In fact, a report of phytocompounds such as hypericin (9.4 mg/L) having a stronger Ki than acarbose (40.6 mg/L) had been reported [40].

*Molecular dynamics simulation*

The binding energy determinations (measured by thermodynamics calculations) is another parameter used as a measure of the fitness of the ligands at the protein’s binding pocket [42]. Higher binding energy depicted by the most negative values is suggestive of a stronger affinity of a ligand for an enzyme, thus, affording better complex stability [43]. The result of the thermodynamic profiles of top compounds of *C. cujete* against each of the studied enzymes is shown in Table 2. A look at these results revealed compounds such as benzoic acid (-48.415 kcal/mol) and phytol (-45.112 kcal/mol) having the most negative binding free energies against alpha-glucosidase which were better than acarbose with -28.248 kcal/mol. The superiority of some plants’ compounds in showing the best complex affinities with their respective enzymes have been seen in some reports [19, 38, 44-45]. Interestingly too, Adinortey et al. [46] also buttressed the superiority of various compounds such as taraxasterol, voruscharin, alpha-amyrin, beta-sitosterol, apigenin-7-0-glucoside, ursolic acid, quercetin-3-rutinoside, and isorhamnetin-3-Orutinoside, from *Calotropis procera* showing better affinity than acarbose (-34.3 kcal/mol) against alpha-glucosidase as depicted by their higher binding energies (ranging from -34.7 kcal/mol to -40.2 kcal/mol). However, with respect to DPP-IV in this study, Diprotin A (standard) had the most negative binding energy value (-45.112 kcal/mol) compared to *C. cujete* compounds, although luteolin (-41.957 kcal/mol), had the most promising affinity out of the five compounds evaluated (Table 2). A similar trend in the superiority of the reference standard (ursolic acid: -18.740 kcal/mol) was also observed against PTP-1B in comparison with *C. cujete* compounds such as naringenin (-16.518 kcal/mol) being the most negative and trans-cinnamic acid (-8.031 kcal/mol), the least negative studied compound. The binding affinity values of the reference standards (Diprotin A and ursolic acid) against DPP-IV and PTP-1B were stronger or the strongest as observed in this study. The observation corroborated the finding from Bower et al. [47] for plant compounds such as hispidulin (-9.4 kcal/mol), naringenin (-8.6 kcal/mol), cirsimaritin (-8.4 kcal/mol), eriodictyol (8.9 kcal/mol) isolated from culinary herbs (*Origanum vulgare*, *Origanum majorana*, *Rosmarinus officinalis*) lower than sitagliptin (-9.6 kcal/mol) used as standard in the study. However, a report of higher negative binding free energy values of some flavonoid c-glycosides such as orientin, vitexin and apigenin above ursolic acid (standard) against PTP1B have also been reported [39]. Against aldose reductase, all the top 5 compounds had higher negative binding free energies compared to ranirestat (-21.042 kcal/mol), with chlorogenic acid (-42.978 kcal/mol) being the most promising compound (Table 2). The observation in this study regarding the affinity of the test compounds for aldose reductase is consistent with a previous study [19], where chlorogenic acid (-41.43 kcal/mol) had a better affinity for the binding pocket of aldose reductase than ranirestat (-38.51 kcal/mol).

Following the establishment of various binding affinities of the compounds and standards with respective targets, there is a need to probe further the extent of stability, flexibility, and compactness of the complex due to the likelihood of a conformational change being imminent as a result of the binding of the ligand which consequently would have an effect on the biological activity of the enzyme [44]. In post-MD simulation, important parameters such as RMSD, RMSF, Rg, and SASA are employed to study the degree of stability or equilibration of the ligand-protein complex [19]. The RMSD measures the change in the dynamics of the protein and the stability (conformation) of the ligand-protein complex [48]. A reduction in the average RMSD value of the complex relative to the apo enzyme during a simulation period is an indication of its stability [49]. Looking at the results of this investigation, the average RMSD of the apo enzyme (alpha-glucosidase) over 100 ns was 1.475 Å which was higher than those of the complexes formed with isoflavone (1.212 Å) and acarbose (1.318 Å) indicating the better stability of these compounds (Table 3). The complex formed with phytol (1.462 Å) showed a marginal decrease (p > 0.05) relative to apo-enzyme (1.475 Å) which is still an indication of the stability of the complex. Isoflavone showing superior stability with its lowest average RMSD value compared to acarbose and other compounds was observed to corroborate the results of thermodynamic profiles. The superior stability of isoflavone-alpha-glucosidase complex over acarbose-alpha-glucosidase was in line with previous studies where natural (phenolic) compounds such as procyanidin, rutin, astilbin depicted lower RMSD values compared to acarbose against alpha-glucosidase [19, 50]. Figure 1a saw the convergence of all the systems at 10 and 20 ns which was maintained over 100 ns except the benzoic acid’s complex which diverged from the alpha-backbone carbon of the enzyme at 20 ns. The average RMSD value of the unbound DPP-IV (1.418 Å) was lower compared with the bound complexes including the standard, DPP-IV-Diprotin A (1.756 Å) and *C. cujete* compounds DPP-IV-xylocaine (1.891 Å), DPP-IV-apigenin (1.905) and DPP-IV-luteolin (2.093 Å) (Table 3). While the superiority of Diprotin A (with marginal decrease compared with cistanoside D, 1.787 Å) in terms of stability above these compounds, as well as their high RMSD values including Diprotin A over the apo enzyme (DPP-IV), was noted, however, such increase has been reported not to be an indication of the complexes’ instability [51-52]. The finding observed in this study was in tandem with reports from Nath et al. [53] and Arif et al. [54] where the RMSD of hit compounds were < 2.5 Å and a convergence of all the systems at 15 ns was also observed before equilibration throughout the simulation period (Figure 1b). 1,2,4,5-tetrazine-3,6-diamine and chlorogenic acid complexes (with PTP-1B) showed the lowest average RMSD values (1.162 Å, 1.242 Å, respectively) followed by PTP-1B-naringenin complex (1.291 Å) compared to the unbound system (1.681 Å). All the bound systems for the compounds revealed a significant (p < 0.05) reduction in average RMSD values (except pinocembrin) compared to the standard, ursolic acid-PTP-1B (1.498 Å) highlighting the significance of their superior structural stability above the standard (Table 3, Figure 1c) and is consistent with a previous observation on RMSD values [55]. A striking fluctuation around 35-40 ns was observed for chlorogenic acid and ursolic acid (Figure 1c). However, this finding differs from the result of the thermodynamic analyses as ursolic acid appeared to show an increased negative score relative to chlorogenic acid, naringenin, and pinocembrin. Although, the variation is noted, however, since MD simulations are used to refine the results emanating from docking scores and are an appropriate tool for providing insight into the evolutionary dynamics of the binding site interaction [56], hence, information on ligand-enzyme complex exhibiting reduced RMSD values relative to the apo enzyme is worth being accorded much importance in terms of consideration or determination of better stability of the complex. Contrary to the RMSD results on alpha-glucosidase and DPP-IV, the *C. cujete* compounds complexation with aldose reductase revealed significant (p < 0.05) lower average RMSD values compared to apo enzyme, aldose reductase (2.126 Å) (Table 3). Interestingly, aldose reductase-ranirestat complex (2.217 Å) was observed to be significantly (p < 0.05) higher compared to the unbound system. While the RMSD of the former is within the acceptable range of 2.5 Å and may not be an indication of instability [51-52], the result was found to be consistent with the binding affinity findings as chlorogenic acid and naringenin with the most negative binding energies were also among the four compounds that formed the best complexes stability with aldose-reductase having a lower average RMSD (1.754, 1.876 Å, respectively), though luteolin and isoflavone were the lowest (1.491 Å, 1.735 Å respectively) as can also be seen in Figure 1d. While it was evident that these compounds promoted good structural stabilities better than the standard (ranirestat) and an indication of their inhibitory role against aldose reductase or being possible drug candidates, the best effect depicted by luteolin in this study was contrary to a previous report [19] with a related compound (luteolin-7-O-beta-D-glucoside) having enhanced negative binding energy (and increased RMSD value) compared to other compounds from *C. edulis* which might be connected to the sugar attachment. Notwithstanding the aforementioned, a study has established good structural stability of naringenin with aldose reductase [57]. Summarily, isoflavone, cistanoside, chlorogenic acid and luteolin were found in this study to reveal the lowest RMSD values often better than respective standards against each of the targets. Since RMSD is a measure of stability [58], hence, the reflection of lower RMSD values of these compounds compared to co-compounds and standard could only be suggested as an indication of better stabilities [19].

In addition to stability, the Rg determines the compactness of the complex [58] and a stable Rg is an indication of stably folded protein [59]. An increased Rg value signifies reduced compactness and vice-versa [60]. The average Rg of apo enzyme (23.531 Å) was significantly (p < 0.05) higher than that of alpha-glucosidase-isoflavone (23.399 Å), alpha-glucosidase-phytol (23.459 Å), and alpha-glucosidase-acarbose (23.486 Å) complexes (Table 3). While the trend of the result is similar to RMSD, the lower Rg values of the complexes with isoflavone and phytol (Figure 2a) relative to acarbose suggest their better compactness and stability since reduced Rg is synonymous to better protein stability [19, 61]. The result of the finding was similar to Eawsakul et al. [50] where astilbin was reported to be more stable with alpha-glucosidase (PDB 5ZCC) based lower Rg value compared to alpha-glucosidase-acarbose. All the complex systems were observed to converge at 10 ns though the higher inconsistency and instability of alpha-glucosidase-benzoic acid complex could be attributed to its higher average Rg value (Figure 2a). However, against DPP-IV, all the bound systems showed a higher average Rg values compared to the unbound system (26.985 Å) with DPP-IV-luteolin complex (27.101 Å) exhibiting the lowest (p < 0.05) increase. The ability of luteolin to compare favourably with Diprotin A (27.118 Å) is a pointer to the superiority of the former above the latter, hence, better compactness (with DPP-IV) and stability. This observation regarding RMSD aligns with thermodynamic energy value results where luteolin had the most negative binding energy compared to Diprotin A. While it was observed that all the systems converge at 30 ns, the high divergence of xylocaine at 10 and 27 ns before stability as a probable reason for the observed increase (including pinocembrin) in average Rg values (Figure 2b). The fluctuation of all the systems against PTP-1B ranges between 18.5 to 19.4 Å and there seems to be a convergence of the systems at around 40 ns (Figure 2c). The average Rg of the unbound PTP-1B was the highest (18.921 Å) compared to the bound systems except trans cinnamic acid (19.043 Å) (Table 3). The marginal decrease in the average Rg value of chlorogenic (18.844 Å) and insignificant increase in the complexes with pinocembrin and 1,2,4,5-tetrazine-3,6-diamine (18.869 Å, 18.871 Å) compared to that of ursolic acid (18.864 Å) may indicate the better stability (with PTP-1B) of the compounds and standard, besides, the ursolic acid may be suggested to be superior going by its highest negative binding energy. The submission in this study contradicts the findings of Rampadarath et al. [39] on the better compactness of flavonoids c-glycosides such as orientin, vitexin, apigenin above ursolic acid. Luteolin-aldose reductase and chlorogenic acid-aldose reductase complexes revealed the lowest average Rg values of 19.165 Å and 19.215 Å, respectively compared to the unbound system, aldose reductase (19.342 Å) and other bound systems [isoflavone-aldose reductase (19.314 Å) aldose reductase-naringenin (19.327 Å)] (Table 3, Figure 2d). The superiority of chlorogenic acid and luteolin in the compactness of the complexes was in line with the results from the thermodynamic energy profiles. The Rg value of the aldose reductase-ranirestat complex (19.357 Å) was the highest among the bound systems, signifying the less stable or instability of its complex relative to the significant and stable compactness of the compounds. While the result follows the same pattern as RMSD, however, the findings buttress the previous submission with *C. edulis* [19] where the Rg of the standard reflected less stability compared to the compounds. Above all, Rg is a function of compactness which partly is also attributed to the stability of the complex; the finding of this study summarily as it concerns Rg depicted isoflavone, luteolin and chlorogenic acid as candidates with better compactness with the four respective targets owing to their lowest Rg values and thus poses better stabilities.

Root mean square fluctuation provides insight to the structural flexibility of the ligand-protein complex [46]. It is a measure of the average displacement of a position during a trajectory period relative to a reference point [62]. An enhanced flexibility movement is concomitant to increased fluctuations existing between the ligands and the amino acids residues of the receptor and vice versa [58]. In this study, the average RMSF value observed with the unbound alpha-glucosidase was higher (1.096 Å) compared to the acarbose-alpha-glucosidase (0.987 Å) complex (Table 3), indicating a lower fluctuation of the complex. Moreover, the average RMSF values of isoflavone-alpha-glucosidase (0.932 Å) and apigenin-alpha-glucosidase (0.956 Å) complexes were lower (p > 0.05) compared to acarbose-alpha-glucosidase system. However, since lower RMSF values indicates better bonding and lesser flexibility of amino acid residues at the binding domain, it could be implicative of little structural distortion [63] and better structural stability [64] of these compounds (isoflavone and apigenin) with alpha-glucosidase and as such, suggest them as better inhibitors of the enzyme than acarbose [50]. While the average RMSF of other compounds such as phytol (1.039 Å) and benzoic acid (1.098 Å) increased the flexibility of the complex, the consistent fluctuations of all the systems were observed at 50, 150, 300 and around 220-240 amino residues with isoflavone showing the lessened fluctuations (Figure 3a) indicating its potential superior possibility of sitting well in the binding pocket of the protein. Against DPP-IV, the average RMSF value of Diprotin A-DPP-IV complex (1.252 Å) was marginally above (p > 0.05) that of the apo enzyme (1.211 Å) suggestive of a possible heightened instability of the complex, however, xylocaine-DPP-IV (1.164 Å), apigenin-DPP-IV (1.182 Å) and cistanoside D (1.187 Å) complexes presented reduced fluctuations lower than Diprotin A indicating lesser flexibilities and as such afforded better stabilities of the complexes. The lower flexibilities observed with these compounds particularly with apigenin (phenolic compound) was in line with findings from a previous study, where chlorogenic acid had lesser flexibility compared to Diprotin A [65]. It was observed that there was a similarity in the pattern of fluctuations for the systems, though, these fluctuations were observed around 20, 50, 150, 200 and 550 amino acids residues (Figure 3b). The importance of these identified residues is that these compounds (xylocaine, apigenin and castanoside D) might possess enhanced effects to maintain a good stability at the enzyme’s receptor site. Against PTP-1B, the apo enzyme depicted a high RMSF value (1.277 Å), this fluctuation was brought down by ursolic acid (1.109 Å) to infer the stability of the complex. Interestingly, the average RMSF of 1,2,4,5-tetrazine-3,6-diamine (0.959 Å) and chlorogenic acid-PTP-1B (0.987 Å) followed by naringenin-PTP-1B (1.076 Å) suggests the superiority of these compounds to provide lesser flexibilities, reduced or no distortions and profound stability. While phenolic compounds such as vitexin and orientin have been reported in Ramparadath et al. [39] study to reveal reduced flexibilities, the lower RSMF values of compounds including naringenin, chlorogenic acid and 1,2,4,5-tetrazine-3,6-diamine from the present work could be said to corroborate earlier study and thus, may be better candidates in the development of probable moieties for diabetes control. Additionally, fluctuations of the systems were observed at 120, 175 and 280 amino acids residues though a reduced fluctuation of the compounds was observed at catalytic region, 175 representing the WPD loop (circled in red) (Figure 3c) as also indicated in the work of Rampadarath et al. [39]. The bound systems revealed a reduction in the fluctuation of the complex when compared to the apo enzyme, aldose reductase with an average RMSF value of 1.089 Å. The average RMSF values of chlorogenic acid-aldose reductase and luteolin-aldose reductase complexes were the lowest (1.022, 1.024 Å respectively) compared to ranirestat-aldose reductase (1.051 Å) and other systems; indicating the potential of the compounds to offer better stability of the complex and thus suggest them as probable candidates for aldose reductase inhibition. The observed effect of chlorogenic and luteolin with reduced distortion by virtue of their lower flexibilities compared to ranirestat aligns with the report of Sabiu et al. [19] where isorhamnetin-3-O-rutinoside and luteolin-7-O-beta-D-glucoside displayed similar characteristics. While fluctuations at 125, 225 amino acids residues were noted for all the systems depicting the highest fluctuation of naringenin (Figure 3d), the observed average higher RMSF of ranirestat relative to lower *C. cujete* phytocompounds in this study was also in tandem with a previous study [19] on *C. edulis* where a related compound, luteolin-7-O-beta-D-glucoside was similarly observed to greatly lessen the flexibility of aldose reductase. Isoflavone, xylocaine, 1,2,4,5-tetrazine-3,6-diamine and chlorogenic acid are compounds with the lowest RMSF values against the studied targets. Lower RMSF values has been reported as indication of lower flexibility and more stability [64]. Hence, with better stabilities of these compounds with respective targets could consider them as likely compound for further drug development.

Solvent accessible surface area determines the degree of hydrophobic exposures of amino acid residues in an effort to predict the stability of the complex [66]. The extent of exposure may cause a conformational change to the protein structure [67]. A decreased SASA value of the bound system relative to the unbound apo enzyme suggests enhanced exposure of the non-polar amino acids residues of the resulting complex, hence conferring better stability [68]. The results obtained with regards to SASA in this study revealed a heightened SASA value with alpha-glucosidase (20486.80 Å), this was brought down to 20389.38 Å with the binding of acarbose indicating the stability of the complex (Table 3). However, apigenin and isoflavone presented the superior stability of the complex beyond acarbose with their lowest average SASA values (19751.53 Å, 19832.15 Å respectively) thus, buttressing their potential as a possible viable candidate in managing postprandial hyperglycaemia. A continuous fluctuation was observed with the apo enzyme over 100 ns, however, the bound systems revealed inconsistent fluctuations (Figure 4a). In a similar trend with alpha-glucosidase, DPP-IV depicted a high SASA value (25134.84 Å) when compared with other bound systems except for apigenin-DPP-IV (25601.66 Å) (Table 3). The ability of the Diprotin A-DPP-IV complex to have a lower SASA value (24891.80 Å) against the apo enzyme was an indication of the complex’s stability. A further reduction was observed with xylocaine, thus, exhibiting its profound stability with Diprotin A. While it was observed that consistency of fluctuations varies in the flexibility of the bound systems throughout the simulation period, that of the apo enzyme (and by extension pinocembrin complex) was found at 30 ns (Figure 4b). The average SASA values for the apoenzymes, PTP-1B and aldose reductase were also hyped (13194.41 Å, 13783.49 Å, respectively) when compared with their complexation with respective standards, ursolic acid and ranirestat (12749.91 Å, 13503.46 Å, respectively). However, the respective bound systems [pinocembrin (12560.01 Å) and luteolin (13116.07 Å)] better stabilized the complexes than the respective standards. The superiority of pinocembrin above ranirestat and luteolin over ursolic acid is an indication that they could serve as possible inhibitors in T2DM and diabetes retinopathy therapies. The fluctuations of the apo enzyme and chlorogenic acid showed consistencies throughout the simulation while other systems were not, though that of pinocembrin (Figure 4c) and luteolin (Figure 4d) and were less flexible. However, ursolic acid only revealed consistent fluctuations against PTP-1B (Figure 4c). Apigenin, xylocaine, pinocembrin and luteolin presented lowest SASA values compared to apo enzymes (alpha-glucosidase, DPP-IV, PTP-1B and aldose reductase respectively) indicating increased exposure of hydrophobic non polar residues which is a consequence of complex stability [68].

The different interactions formed in complexes arising from the binding of compounds (standard drugs and *C. cujete* compounds) to the investigated enzymes are presented in Table 4 and Supplementary Figure S1. The nature of interactions (type and/ or number) existing between the ligand and the enzyme presents insight into the degree of affinity [69-71]. Additionally, the interaction (bond) between the ligand and the respective targets has an influence on the binding free energy of the complex [72]. In this study, the interactions existing between the top compounds of *C. cujete* and respective targets are summarized in Table 4. Looking at the results, acarbose had the highest interactions (30) comprising 20 van der Waal forces, 3 H-bonds, 1 C-H bond, 1 unfavourable donor-donor bond and 1 unfavourable bump in its complexation with alpha-glucosidase (Figure 5a) as compared to *C. cujete* compounds [phytol (23), apigenin (16), isoflavone (16) and benzoic acid (15)]-alpha-glucosidase complexes (Table 4). The number of interactions observed from each of the complexes did not translate or conform with the report of the binding affinity as benzoic acid (-48.414 Å) with the most negative binding affinity depicted the lowest number of interactions (15) (Figure 5b) whereas, acarbose with a moderate binding affinity (-28.248 Å) in comparison with other systems showed the highest interactions (30). However, a view at the bond length revealed the bond distance between H, with O atoms and amino acid residues (Asn328, Gly225) of benzoic acid to be shorter thus, suggesting its stability; a shorter bond length has been reported to contribute to the stability of the complex than longer bond length [73]. While acarbose depicted a high number of interactions, the presence of unfavourable (bump and donor-donor) bonds might be suggested as probable a reason for its moderate binding affinity or lower binding affinity (compared to benzoic acid and phytol). Similarly, H-bonds contribute to higher energies to the complex [46], hence, the lower binding affinity may suggest the lack of contribution of these H- bonds residues (Asn301, Asp202, Arg200, Gln170) to the complex [46]. Against DPP-IV, luteolin and xylocaine had 15 interactions each [luteolin (3 H-bonds, 11 van der Waals, and 1 π-π stacked), xylocaine (5 H-bonds, 8 van der waal, 1 unfavourable donor-donor and 1 unfavourable acceptor-acceptor bonds) which was the highest followed by pinocembrin [(12); 3 H-bonds, 8 van der Waals forces 1 π-sigma bond], Diprotin A (2 H-bonds, 3 van der Waals, 3 π-alkyl, 2 salt bridges and 1 attractive charge) and castanoside (4 H-bonds, 6 van der Waals, 1 π-alkyl) with 11 interactions (Table 4). Although, luteolin and xylocaine have an equal number of interactions but their energy profiles differ with luteolin having increased negative binding energies (Table 2) which could be attributed to the higher number of important interactions such as conventional H and carbon H-bonds (Figure 5c) since the presence of important interactions have been reported to present higher negative binding energy [46, 69]. However, the highest binding affinity of Diprotin A (-45.112 Å) which was not justified by the fewer number of interactions (Figure 5d) could be indicative of a variety of interaction types (H-bonds, van der Waal, π-alkyl bonds, salt bridge, unattractive charges) present. While it could be noted that the two H bonds could have been responsible for increased binding affinity recorded, at the same time electrostatic interaction such as H-N-H formed with amino acid residues (Glu168, Glu169) on the complex may also influence the binding affinity of the complex [74]. Ursolic acid (Figure 5e) and trans-cinnamic acid (Figure 5f) formed 13 interactions each against PTP-1B (Table 4). The increased number of interactions of ursolic acid (which was not only corroborated by the binding energy profile) made it superior to trans-cinnamic acid and could be attributed to a higher number of π bonds as the presence of π-bond or interaction between amino acids residues, especially π-π stacked interactions have been reported to influence higher bond energy [19] and involved in drug development [75]. Naringenin came next with 10 interactions which also confirms its next higher negative binding energy. For aldose reductase, the complex formed with luteolin had 25 interactions (9 H-bonds, 14 van der Waals, 1 π-π stacked, 1 π-alkyl), this was followed by chlorogenic acid-aldose reductase [20 (7 H-bonds, 11 van der Waals, 1 π-π T-shaped, 1 π-alkyl)], naringenin-aldose reductase (16) and the standard, ranirestat-aldose reductase (11) (Table 4). While the result of the interaction plots was in tandem with the finding of energy profiles between the standard and the compounds, however, the complex with chlorogenic acid with the highest binding free energy values revealed a lesser number of interactions as compared to luteolin (Figure 5g) with a higher number of interactions and reduced negative energies. This observation could be said to be attributed to several factors such as probable failure of the H-bonds responsible for contributing energies to the complex for higher binding affinity to do so [46], bond lengths, other unfavourable bonds and lack of interaction with important catalytic amino acids residues such as Tyr48 and His110. Additionally, the increased number of interactions for chlorogenic acid-aldose reductase complex over ranirestat-aldose reductase interactions (Figure 5h) is consistent with a previous report [19], where chlorogenic acid had 16 interactions as compared to ranirestat with 14 interactions.

*Pharmacokinetics*

The ADME properties prediction of a compound gives an idea of its profiles in terms of bioavailability and toxicity when considered as a drug for possible development [76]. In determining the pharmacological profiles and coming to a decision on the prospective bioactive drug moiety, Lipinski’s rule of 5 which explores the molecular weight (> 500 kilo Dalton), number of H donor (less or equal to 5), number of H acceptor (less or equal to 10) and octanol coefficient (less than 5) is usually employed. Based on the result of this investigation which looked at the overall 14 best compounds and respective standards, 12 of the compounds passed Lipinski’s rule while castanoside D and catalposide failed with acarbose (which is unexpectedly being a standard drug) (Table 5). The implication is that these 12 compounds have the potentials to be able to pass through the systemic circulation (unhindered) to elicit their pharmacological action as drug candidates [77]. Since the rate of absorption and amount of unchanged drug passing the blood is assessed by their bioavailability score (BS), this submission on Lipinski’s rule was corroborated by the BS, where 11 of the compounds except chlorogenic acid (11%), cistanoside D (17%) and catalposide (17%) had bioavailability scores above 20%. While benzoic acid was best bioavailable (85%) and the minimally acceptable bioavailability limit for a drug candidate is 10% [78], it then means that all the 14 compounds have worthy profiles as probable candidates in the management of T2DM though it must be noted that ursolic acid among the reference standards was poorly absorbed. In terms of aqueous solubility and gastrointestinal absorption, all the 14 compounds and standards revealed varying levels of solubility, however, chlorogenic acid, castanoside D, catalposide, glucopyranoside, phytol among the test compounds and acarbose as well as ursolic acid presented low GI absorption. This could mean that these compounds (chlorogenic acid, castanoside D, catalposide, glucopyranoside, phytol) might require further optimization or modification for them to serve as good inhibitors to targets of T2DM, besides, none of these promising candidates against aldose reductase are poorly GI absorbed. Apigenin, isoflavone and pinocembrin among the 14 compounds inhibited at most 2 of the cytochrome isoenzymes while others were observed not to inhibit four or five of the isoenzymes (Table 5). Cytochrome isoenzymes are key in drug metabolism and the ability of seven (benzoic acid, castanoside D, catalposide, glucopyranoside, chlorogenic acid, trans cinnamic acid and 1,2,4,5- tetrazine- 3,6 diamine) of the compounds not to inhibit all the cytochromes signifies their potential safety (in causing drug-drug toxicity) and superiority over others [64].

The use of alternative therapeutic medicine with natural products is considered a laudable approach in the management of T2DM and its related complication such as diabetic retinopathy. Since alpha-glucosidase, PTP-1B and DPP-IV as well aldose reductase are prominent targets for effective treatment of T2DM and diabetic retinopathy respectively, the identification and discovery of isoflavone (based on all parameters checked in this study) observed to form or maintain good stability with alpha-glucosidase, and xylocaine as well as chlorogenic acid against DPP-IV and PTP-1B, respectively preferred them as ideal candidates from *C. cujete* towards T2DM therapy. Additionally, with luteolin replicating similar effect as isoflavone, xylocaine and chlorogenic acid, it may also be handy in the development of a good drug in the management of diabetic retinopathy.

**Conclusion**

The prevalence of T2DM is concerning and the hallmark in its management is towards regulating the blood glucose to normalcy which since time immemorial are managed with different classes of OHA influencing the key carbohydrate-metabolizing enzymes. While the use of synthetic drugs has largely undermined any side effects, consideration of alternative options in medicinal plants such as *C. cujete* with established antidiabetic effects is welcoming and laudable, though, the implicated phytocompounds is/are wanting or yet to be discovered. The present study explored the therapeutic action of various identified compounds from *C. cujete* as probable therapeutic drug candidates through computational studies. Based on the findings from thermodynamic profiles, MD and post-MD simulation metrics, binding interactions and pharmacokinetic profiles of each of the compounds with the respective target, the study concludes that compounds such as isoflavone (against alpha-glucosidase), xylocaine (DPP-IV), chlorogenic acid (PTP-1B) and luteolin (aldose reductase) as promising inhibitors of the studied carbohydrate-metabolizing enzymes. Hence, exploiting them as novel drug moieties in the management of T2DM and its related retinopathy complication (luteolin) would go a long way in drastically reducing the disorder's continuous emergence.

**Acknowledgement**

The authors appreciate the postdoctoral fellowship awarded to Dr FO Balogun by National Research Foundation of South Africa, tenable at Department of Biotechnology and Food Technology, DUT, South Africa. The authors appreciate Research and Development of the Durban University of Technology (DUT), Durban, University for the emerging researcher’s grant awarded to Dr FO Balogun

**Competing Interest**

No conflicting interest among the authors

**References**

[1] Sapra A, Bhandari P. Diabetes Mellitus. [Updated 2021 Sep 18]. In: StatPearls [Internet]. Treasure Island (FL): StatPearls Publishing. https://www.ncbi.nlm.nih.gov/books/NBK551501/. Accessed 20 Jan 2022.

[2] WHO. Diabetes. https://www.who.int/news-room/fact-sheets/detail/diabetes. Accessed Dec 13 2021

[3] Westman EC. Type 2 Diabetes Mellitus: A Pathophysiologic Perspective. Front Nutr. 2021;8.https://doi=10.3389/fnut.2021.707371

[4] American Diabetes Association. Diagnosis and classification of diabetes mellitus. 2011

[5] Cho NH, Shaw JE, Karuranga S. IDF diabetes atlas: Global estimates of diabetes prevalence of for 2017 and projections for 2045. J Diab. 2018;138: 271-28.

[6] Ali MY, Jannat S, Rahman MM. Investigation of C-glycosylated apigenin and luteolin derivatives’ effects on protein tyrosine phosphatase 1B inhibition with molecular and cellular approaches. Comput Toxicol. 2020; doi: https://doi.org/10.1016/j.comtox.2020.100141

[7] Dodds S. The how-to-for type 2: An overview of diagnosis and management of type 2 diabetes mellitus. Nurs Clin North Am. 2017;52: 513-522

[8] Kazmi M, Zaib S, Ibrar A, Amjad ST, Shafique Z, Mehsud S, et al. A new entry into the portfolio of α-glucosidase inhibitors as potent therapeutics for type 2 diabetes: Design, bioevaluation and one-pot multi-component synthesis of diamine-bridged coumarinyl oxadiazole conjugates. Bioorg Chem. 2018;77:190–202.

[9] Liu S-K, Hao H, Bian Y, Ge Y-X, Lu S, Xie H-X, Wang K-M, Tao H, Yuan C, Zhang J, Zhang J, Jiang C-S and Zhu K. Discovery of new α-glucosidase inhibitors: structure-based virtual screening and biological evaluation. Front Chem. 2021;9:639279. https://doi.10.3389/fchem.2021.639279

[10] Rosa MM, Dias T. Commonly used endocrine drugs. Edited by José Biller, José M. Ferro. In: Handbook of Clinical Neurology. Elsevier Publishers; 2014;120:809-824

[11] Huang PK, Lin SR, Chang CH, *et al.* Natural phenolic compounds potentiate hypoglycemia via inhibition of Dipeptidyl peptidase IV. Sci Rep. 2019;9:15585. https://doi.org/10.1038/s41598-019-52088-7

[12] Zhao BT, Le DD, Nguyen PH, Ali My, Choi JS, Min BS, Shin HN, Rhee HI, Woo MH. PTP1B, alpha-glucosidase and DPPIV inhibitory effects of chromene derivatives from the leaves of *Smilax china* L. Chem Biol Interact. 2016;25(253):27-37

[13] Antonetti DA, Klein R, Gardner TW. Diabetic retinopathy. N Engl J Med. 2012;366(13):1227–1239

[14] Duh EJ, Sun JK, Stitt AW. Diabetic retinopathy: current understanding, mechanisms, and treatment strategies. JCI Insight. 2017;2(14):e93751. https://doi.org/10.1172/jci. insight.93751

[15] Mohammed Q, Gilles MC, Wong TY. Management of diabetes retinopathy: A systematic Review. J Am Med Assoc. 2017;208:902- 916

[16] Karasu C, Cumao˘ glu A, Gürpinar AR, Kartal M, Kovacikova L, Milackova I Stefek M. Aldose reductase inhibitory activity and antioxidant capacity of pomegranate extracts. Interdiscip Toxicol. 2012;5:15–20.

[17] Sabiu S, O’Neill FH, Ashafa AOT. Kinetics of α-amylase and α-glucosidase inhibitory potential of Zea mays Linnaeus (Poaceae), *Stigma maydis* aqueous extract: An *in vitro* assessment. J Ethnopharmacol. 2016;183: https://doi.org/10.1016/j.jep.2016.02.024.

[18] Balogun FO, Ashafa AOT. Aqueous root extract of *Dicoma anomala* (Sond.) extenuates postprandial hyperglycaemia *in vitro* and its modulation on the activities of carbohydrate-metabolism enzymes in streptozotocin –induced diabetic Wistar rats. S Afr J Bot. 2017;112:102-112.

[19] Sabiu S, Balogun FO, Amoo SO. Phenolics Profiling of *Carpobrotus edulis* (L.) NE Br. and Insights into molecular dynamics of their significance in type 2 diabetes therapy and its retinopathy complication. Mol. 2021;26: 4867.

[20] Ebrahimie M, Bahmani M, Shirzad H, Rafieian-Kopaei M, Saki K. A Review Study on the effect of Iranian herbal medicines on Opioid withdrawal syndrome. J Evid Based Complementary Altern Med. 2015;20: 302-309.

[21] Bahmani M, Rafieian-Kopaei M, Naghdi N, Nejad ASM, Afsordeh O. *Physalis alkekengi*: A review of its therapeutic effects. J Chem Pharm Sci. 2016;9(3): 1472-1485.

[22] Mozaffari Nejad AS, Kamkar A, Giri A, Pourmahmoudi AA. Ethnobotany and folk medicinal uses of major trees and shrubs in Northern Iran. J Med Plants Res. 2013;7: 284-289.

[23] Bahmani M, Shirzad H, Rafieian S, Rafieian Kopaei M. Silybum marianum: Beyond Hepatoprotection. Evid J Based Complementary Altern Med. 2015;20: 292-301.

[24] Balogun FO, Sabiu S. A review of the phytochemistry, ethnobotany, toxicology, and pharmacological potentials of *Crescentia cujete* L. (Bignoniaceae). Evidence-Based Complement Altern Med. 2021; 6683708, 15 pages. https://doi.org/10.1155/2021/6683708

[25] Parente FGG, de Oliveira AP, Rodrigues CMSC. Phytochemical screening and antioxidant activity of methanolic fraction from the leaves of *Crescentia cujete* L. (Bignoniaceae). J Chem Pharm Res. 2017;8(2):231–236.

[26] Iwueke AV, Nwodo OFC. Antihyperglycaemic effect of aqueous extract of *Daniella oliveri* and *Sarcocephalus latifolius* roots on key carbohydrate metabolic enzymes and glycogen in experimental diabetes. Biokemistri. 2008;20 (2)

[27] Atolani O, Olatunji GA. Isolation and evaluation of antiglycation potential of polyalthic acid (furano-terpene) from *Daniella oliveri*. J Pharm Anal. 2014;4(6):407–411.

[28] Shauibu G, Abu AH, Agwu EO, Ehile V, Ezeudo ZE, Ogedemgbe B, Peter S. Effects of *Daniella oliveri* aqueous leaves extract on blood glucose, haematological and biochemical values in streptozotocin-induced type 2 diabetes in male albino rats. Vom J Vet Sci. 2018;13(1):81-87

[29] Adeyemi SB, Surana V, Krishnamurthy R. In-vitro inhibition of type 2 diabetes key enzymes; effect of extracts and solvent-solvent fractions of *Daniellia oliveri* (Rolfe) Hutch. & Dalziel. Bull Pharm Sci Assiut Uni. 2021;44(2):415-426

[30] Semporé JN, Diao M, Ouattara L, Ouoba P, Kagambega W, Sama H, Dibala CI, Konaté K, Dicko MH. Potential antidiabetic effects of extracts from four medicinal plants used in Burkina Faso by inhibition of alpha-amylase. Diab. 2021;2(4):250-258.

[31] Branden G, Sjogren T, Schnecke V, Xue Y. Structure-based ligand design to overcome CYP inhibition in drug discovery projects. Drug Discov Today 2014;19: 905–911.

[32] Ambrose GO, Afees OJ, Nwamaka NC, Simon N, Oluwaseun AA, Soyinka T, Bankole S. Selection of Luteolin as a potential antagonist from molecular docking analysis of EGFR mutant. Bioinform. 2018;14:241.

[33] Ali DM, Saalis JM, Sathya R, Irfan N, Kim JW. An evidence of microalgal peptides to target spike protein of COVID-19: *in silico* approach, Microb Pathog. 2021;160:105189. https://doi.org/10.1016/j. micpath.2021.105189

[34] Ortiz CLD, Completo GC, Nacario RC *et al.* Potential inhibitors of Galactofuranosyltransferase 2 (GlfT2): molecular docking, 3D-QSAR, and *In silico* ADMETox Studies. *Sci Rep. 2019;* 9:17096. https://doi.org/10.1038/s41598-019-52764-8

[35] Seifert E. Origin Pro 9.1: Scientific Data Analysis and Graphing Software-Software Review. J Chem Informat Modeling 2014;54. 10.1021/ci500161d.

[36] Sliwoski G, Kothiwale S, Meiler J, Lowe Jr EW. Computational methods in drug discovery. Pharmacol Rev. 2014;66(1):334-395

[37] Salmaso V, Moro S. Bridging molecular docking to molecular dynamics in exploring ligand-protein recognition process: An overview. Front Pharmacol. 2018;9. https://.doi.10.3389/fphar.2018.00923

[38] Akinyede KA, Oyewusi HA, Hughes GD, Ekpo OE, Oguntibeju OO. In vitro evaluation of the anti-diabetic potential of aqueous acetone *Helichrysum petiolare* extract (AAHPE) with molecular docking relevance in diabetes mellitus. Mol. 2021;27(1):155. https://.doi.10.3390/molecules27010155.

[39] Rampadarath A, Balogun FO, Charlene P. Sabiu S. Identification of flavonoid c-glycosides as promising antidiabetics targeting protein tyrosine phosphatase 1B. J Diab Res. 2022;6233217. https://doi.org/10.1155/2022/6233217

[40] Dong Q, Hu N, Yue H, Wang H. Inhibitory activity and mechanism investigation of Hypericin as a novel *α*-Glucosidase inhibitor. Mol. 2021;26(15):4566. doi: 10.3390/molecules26154566

[41] Pan J, Zhang C, Yang W, Liu H, Lv Z, Liu J and Jiao Z. Inhibition of dipeptidyl peptidase-4 by flavonoids: structure-activity relationship, kinetics and interactions mechanism. Front Nutr. 2022;9:892426. Doi.10.3389/fnut.2022.892426

[42] Decherchi S, Cavalli A. Thermodynamics and kinetics of drug-target binding by molecular simulation. Chem Rev. 2020;120(23):12788-12833. doi: 10.1021/acs.chemrev.0c00534.

[43] Zabidi NA, Ishak NA, Hamid M, Ashari SE, Mohammad Latif MA. Inhibitory evaluation of *Curculigo latifolia* on glucosidase, DPP (IV) and *in vitro* studies in antidiabetic with molecular docking relevance to type 2 diabetes mellitus. J Enzym Inhib Med Chem. 2021;36: 109–121.

[44] Sindhu R, Binod P, Pandey A. Biological pretreatment of lignocellulosic biomass—An overview. Bioresour Technol. 2015;199:76–82.

[45] Salim B, Said G, Kambouche N, Kress S. Identification of phenolic compounds from Nettle as new candidate inhibitors of main enzymes responsible on type-II diabetes. Curr Drug Discov Technol. 2020;17:197.

[46] Adinortey CA, Kwarko GB., Koranteng R., Boison D, Obuaba I, Wilson MD, Kwofie SK. Molecular structure-based screening of the constituents of *Calotropis procera* identifies potential inhibitors of diabetes mellitus target alpha glucosidase. Curr Issues Mol Biol. 2022;44:963–987. https://doi.org/ 10.3390/cimb44020064

[47] Bower AM, Real Hernandez LM, Berhow MA, de Mejia EG. Bioactive compounds from culinary herbs inhibit a molecular target for type 2 diabetes management, dipeptidyl peptidase IV. J Agric Food Chem. 2014;62(26): 6147–6158.

[48] Muralidharan N, Sakthivel R, Velmurugan D, Michael Gromiha M. Computational studies of drug repurposing and synergism of lopinavir, oseltamivir and ritonavir binding with SARS-CoV-2 protease against COVID-19. J Biomolec Struc Dyn. 2020. https://.doi.10.1080/07391102.2020.1752802

[49] Childers MC, Daggett V. Insights from molecular dynamics simulations for computational protein design. Mol Syst Des Eng. 2017;12(1): 9-33

[50] Eawsakul K, Ongtanasup T, Ngamdokmai N. *et al.* Alpha-glucosidase inhibitory activities of astilbin contained in *Bauhinia strychnifolia* Craib. stems: an investigation by in silico and in vitro studies. *BMC Complement Med Ther.* 2023;23:25. https://doi.org/10.1186/s12906-023-03857-5

[51] Rosenberg MS. Sequence alignment: methods, models, concepts and strategies. Bioinform 1972. https:doi:10.1525/9780520943742

[52] Fusani L, Palmer DS, Somers DO, Wall ID. Exploring ligand stability in protein crystal structures using binding pose metadynamics. J Chem Inf Model. 2020;60(3):1528-1539.

[53] Nath V, Manish R, Neeraj K, et al. Computational identification of potential dipeptidyl peptidase (DPP)-IV inhibitors: Structure based virtual screening, molecular dynamics simulation and knowledge-based SAR studies. J Mole Struct. 2021;129006(2021). https://doi:10.1016/j.molstruc.2020.129006

[54] Arif R, Ahmad S, Mustafa G, Mahrosh HS, Ali M, Qamar MT, Dar HR. Molecular docking and simulation studies of antidiabetic agents devised from hypoglycemic polypeptide-P of *Momordica charantia*. BioMed Res Int. 2021;5561129. https://doi.org/10.1155/2021/5561129

[55] Martinez L. Automatic Identification of Mobile and Rigid Substructures in Molecular Dynamics Simulations and Fractional Structural Fluctuation Analysis. PLOS One 2015;10(3).

[56] Antony P, Vijayan R. Identification of novel aldose reductase inhibitors from spices: A molecular docking and simulation study. PLoS One. 2018;10(9):e0138186. doi: 10.1371/journal.pone.0138186.

[57] Khan MS, Qais FA, Rehman M, Ismail MH, Alokail MS, Altwaijry N, Alafaleq NO, AlAjmi MF, Salem N, Alqhatani R. Mechanistic inhibition of non-enzymatic glycation and aldose reductase activity by naringenin: Binding, enzyme kinetics and molecular docking analysis. Int J Biol Macromol. 2020;159:87-97

[58] Shode FO, Idowu ASK, Uhomoibhi OJ, Sabiu S. Repurposing drugs and identification of inhibitors of integral proteins (spike protein and main protease) of SARS-CoV-2. J Biomol Struct Dyn. 2021.

[59] Galzitskaya OV, Garbuzynskiy SO. Entropy capacity determines protein folding. Proteins Struct Funct Genet. 2006;63:144–154.

[60] Cherrak SA, Merzouk H, Mokhtari-Soulimane N. Potential bioactive glycosylated flavonoids as SARS-CoV-2 main protease inhibitors: A molecular docking and simulation studies. PLoS ONE 2020;15(10): e0240653. https://doi.org/10.1371/journal.pone.0240653

[61] Chen J, Wu S, Zhang Q, Yin Z, Zhang L. α-Glucosidase inhibitory effect of anthocyanins from *Cinnamomum camphora* fruit: Inhibition kinetics and mechanistic insights through in vitro and in silico studies. Int J Biol Macromol. 2020;143:696–703

[62] Cholko T, Chen W, Tang Z, Chang C. A molecular dynamics investigation of CDK8/CycC and Ligand binding: Conformational flexibilty and implication in drug discovery. J Comput Aided Mol Des. 2017;176:139–148

[63] Jin Z, Du X, Xu Y, Deng Y, Liu M, Zhao Y, Zhang B, Li X, Zhang L, Peng C, Duan Y, Yu J, Wang L, Yang K, Liu F, Jiang R, Yang X, You T, Liu X, et al., Structure of Mpro from SARS-CoV-2 and discovery of its inhibitors. Nature 2020; 582(7811): 289–293.

[64] Aribisala JO, Abdulsalam RA, Dweba Y, Madonsela K, Sabiu S. Identification of secondary metabolites from *Crescentia cujete* as promising antibacterial therapeutics targeting type 2A topoisomerases through molecular dynamics simulation. Comp Biol Med. 2022;145.105432.

[65] Balogun FO, Naidoo K, Aribisala JO, Pillay C, Sabiu S. Cheminformatics identification and validation of dipeptidyl peptidase-IV modulators from shikimate pathway-derived phenolic acids towards interventive type-2 diabetes therapy. Metabolites 2022; 12:937. <https://doi.org/> 10.3390/metabo12100937

[66] Khan S, Bjij I, Betz RM, et al. Reversible versus irreversible inhibition modes of ERK2: A comparative analysis for ERK2 protein kinase in cancer therapy. Future Med Chem 2018;10(9):1003–1015

[67] Zhang D, Lazim R. Application of conventional molecular dynamics simulation in evaluating the stability of apomyoglobin in urea solution. Scientif Rep. 2017;7(44651).

[68] Khan S, Fakhar Z, Hussain A, Ahmad A, Jairajpuri D, Alajmi M, Hassan M. Structure-based identification of potential SARS-CoV-2 main protease inhibitors. J Biomolec Struct Dyn. 2020;1-14.

[69] Chen J, Almo SC, Wu Y. General principles of binding between cell surface receptors and multi-specific ligands: A computational study. PLoS Comput Biol. 2017;13(10):e1005805. https://.doi: 10.1371/journal.pcbi.1005805.

[70] Kairys V, Baranauskiene L, Kazlauskiene M, Matulis D, Kazlauskas E. Binding affinity in drug design: Experimental and computational techniques. Expert Opin. Drug Discov. 2019;14:755–768.

[71] Vergara R, Romero-Romero S, Velázquez-López I, et al. The interplay of protein-ligand and water-mediated interactions shape affinity and selectivity in the LAO binding protein. FEBS J 2020;287(4):763-782; doi: 10.1111/febs.15019.

[72] Izadi H, Stewart KME, Penlidis A, Role of contact electrification and electrostatic interactions in gecko adhesion, J R Soc Interface 2014;11:371–398

[73] Du X, Li Y, Xia YL, Ai SM, Liang J, Sang P, Ji XL, Liu SQ. Insights into protein-ligand interactions: mechanisms, models, and methods. Int J Mol Sci. 2016;17(2):144. https://doi:10.3390/ijms17020144.

[74] Bayas MV, Kearney A, Avramovic A, Anton van der Merwe P, Leckband DE. Impact of salt bridges on the equilibrium binding and adhesion of Human CD2 and CD58. J Biol Chem. 2007;282(8):5589 –5596

[75] Babine RE, Bender SL. Molecular recognition of protein-Ligand complexes: Applications to drug design. Chem Rev. 1997; 97:1359–1472.

[76] Yamashita F, Hashida M, *In silico* approaches for predicting ADME properties of drugs, Drug Metabol Pharmacokinet. 2004;19:327–338.

[77] Remko M, Boh A, Kov´aˇcikov´a L, Molecular structure, pKa, lipophilicity, solubility, absorption, polar surface area, and blood brain barrier penetration of some antiangiogenic agents, Struct Chem. 2011;22:635–648

[78] Price G, Patel DA. Drug Bioavailability 2020

Table 1. Docking scores of the best five compounds against each of the diabetic target enzymes

| **Targets** | **Compound/standards** | **Binding energy (kcal/mol)** | **Ki (µM)** |
| --- | --- | --- | --- |
| Alpha-glucosidase^#^ | Isoflavone | -7.7 | 0.05 |
|  | Phytol | -7.4 | 0.06 |
|  | Apigenin | -7.3 | 0.06^^^ |
|  | Benzoic acid | -7.2 | 0.06^^^ |
|  | Acarbose* | -5.0 | 0.14 |
| Dipeptidyl peptidase-IV | Luteolin | -6.7 | 0.07 |
|  | Xylocaine | -5.7 | 0.11 |
|  | Apigenin | -5.7 | 0.11 |
|  | Pinocembrin | -5.7 | 0.11 |
|  | Cistanoside D | -5.7 | 0.11 |
|  | Diprotin A* | -5.5 | 0.12 |
| Protein Tyrosine Phosphatase-1B | Chlorogenic acid | -6.2 | 0.10 |
|  | 1,2,4,5-tetrazine-3.6-diamine | -5.9 | 0.10 |
|  | Pinocembrin | -5.9 | 0.10 |
|  | Naringenin | -5.7 | 0.11 |
|  | Trans-cinnamic | -5.7 | 0.11 |
|  | Ursolic acid* | -7.4 | 0.06 |
| Aldose reductase^#^ | Naringenin | -9.9 | 0.02 |
|  | Luteolin | -9.7 | 0.02^^^ |
|  | Isoflavone | -9.6 | 0.02^^^ |
|  | Chlorogenic acid | -9.4 | 0.03 |
|  | Ranirestat* | -9.0 | 0.03^^^ |

*Reference standards; ^#^Four compounds selected against alpha-glucosidase and aldose reductase because many compounds have same binding energy scores (-7.1 and -8.7 kcal/mol respectively) for the next lower docking scores; ^^^Values after rounding up to 2 decimal places

Table 2. Thermodynamic profiles of the top compounds with the target enzymes

| **Energy components (kcal/mol)** | | | | | |
| --- | --- | --- | --- | --- | --- |
| **Complexes** | **ΔE_vdW_** | **ΔE_elec_** | **ΔG_gas_** | **ΔG_solv_** | **ΔG_bind_** |
| **Alpha-glucosidase** | | | | | |
| AG + Acarbose | -37.431 ± 5.05 | -39.417 ± 12.88 | -66.466 ± 12.09 | 26.613 ± 6.42 | -28.248 ± 3.35 |
| AG + Apigenin | -39.997 ± 2.49 | -6.959 ± 7.80 | -46.956 ± 6.89 | 20.807 ± 4.91 | -26.150 ± 3.82 |
| AG + Benzoic acid | -49.945 ± 3.47 | -56.287 ± 9.72 | -106.232 ± 9.38 | 57.818 ± 7.27 | -48.415 ± 4.44 |
| AG + Isoflavone | -33.307 ± 2.16 | -4.994 ± 3.49 | -38.300 ± 4.40 | 11.227 ± 2.60 | -27.073 ± 3.18 |
| AG + Phytol | -48.510 ± 3.91 | -8.244 ± 4.84 | -56.754 ± 6.49 | 11.643 ± 2.25 | -45.112 ± 6.10 |
| **DPP-IV** | | | | | |
| DPP-IV + Diprotin A | -48.509 ± 3.91 | -8.244 ± 4.84 | -56.754 ± 6.49 | 11.642 ± 2.24 | -45.112 ± 6.10 |
| DPP-IV + Xylocaine | -45.001 ± 4.50 | -30.372 ± 14.24 | -75.373 ± 14.09 | 49.707 ± 9.61 | -25.666 ± 6.29 |
| DPP-IV + Pinocembrin | -20.909 ± 3.00 | -22.914 ± 8.61 | -43.824 ± 9.16 | 25.344 ± 7.01 | -18.480 ± 3.74 |
| DPP-IV + Luteolin | -34.908 ± 3.77 | -54.367 ± 15.81 | -89.274 ± 16.55 | 47.317 ± 11.98 | -41.957 ± 6.49 |
| DPP-IV + Cistanoside D | -41.517 ± 5.55 | -24.131 ± 17.09 | -65.648 ± 19.14 | 44.739 ± 14.41 | -20.909 ± 7.29 |
| DPP-IV + Apigenin | -25.667 ± 3.91 | -32.933 ± 7.53 | -58.600 ± 7.76 | 34.741 ± 4.70 | -23.859 ± 5.15 |
|  |  |  | **PTP-1B** |  |  |
| PTP-1B + Ursolic acid | -30.044 ± 4.12 | -8.593 ± 6.10 | -31.584 ± 7.53 | 12.844 ± 4.36 | -18.740 ± 5.25 |
| PTP-1B + Chlorogenic acid | -24.702 ± 4.05 | -22.648 ± 12.89 | -47.349 ± 14.51 | 31.421 ± 10.80 | -15.928 ± 5.11 |
| PTP-1B + TZD | -12.423 ± 4.67 | -18.396 ± 9.01 | -30.820 ± 12.41 | 20.161 ± 8.24 | -10.658 ± 4.88 |
| PTP-1B + Naringenin | -22.280 ± 6.51 | -12.275 ± 8.66 | -34.555 ± 12.69 | 18.037 ± 6.86 | -16.518 ± 7.03 |
| PTP-1B + Pinocembrin | -22.116 ± 5.21 | -7.880 ± 6.84 | -30.001 ± 9.93 | 14.628 ± 5.27 | -15.3721 ± 5.69 |
| PTP-1B + TCA | -9.1595 ± 8.50 | -7.275 ± 9.05 | -16.434 ± 16.23 | 8.403 ± 8.11 | -8.031 ± 8.47 |
| **Aldose reductase** | | | | | |
| AR + Ranirestat | -30.729 ± 4.81 | -10.134 ± 6.41 | -40.863 ± 7.04 | 19.821 ± 4.56 | -21.042 ± 3.83 |
| AR + Chlorogenic acid | -41.940 ± 3.72 | -48.666 ± 16.38 | -90.606 ± 16.62 | 47.628 ± 7.83 | -42.978 ± 9.97 |
| AR + Isoflavone | -30.129 ± 7.32 | -6.039 ± 4.66 | -36.168 ± 9.46 | 13.280 ± 4.19 | -22.887 ± 6.20 |
| AR + Luteolin | -54.803 ± 3.88 | -6.784 ± 14.19 | -26.587 ± 13.13 | 16.378 ± 7.60 | -30.209 ± 6.94 |
| AR + Narengenin | -39.275 ± 2.38 | -10.957 ± 7.90 | -50.232 ± 7.40 | 18.940 ± 4.73 | -31.292 ± 3.94 |

AG-Alpha-glucosidase; AR: Aldose reductase; PTP-1B: protein tyrosinase phosphatase; DPP-IV: dipeptidyl peptidase-IV; TZD: 1,2,4,5-tetrazine-3,6-diamine; TCA: trans cinnamic acid; ΔEvdW = van der Waals; ΔEelec = electrostatic energy; ΔGgas = gas phase free energy; ΔGsolv = solvation free energy; ΔGbind = total binding energy

Table 3. Average post-dynamic simulation parameters of the top compounds with the carbohydrate metabolizing enzymes

| **Complexes** | **RMSD** (Å) | **Rg** (Å) | **RMSF** (Å) | **SASA** (Å) |
| --- | --- | --- | --- | --- |
| AG | 1.475 ± 0.26^a^ | 23.531 ± 0.12^a^ | 1.096 ± 0.47^a^ | 20486.80 ± 7.15^a^ |
| AG + Acarbose | 1.318 ± 0.17^b^ | 23.486 ± 0.09^b^ | 0.987 ± 0.44^b^ | 20389.38 ± 5.79^b^ |
| AG + Isoflavone | 1.212 ± 0.10^c^ | 23.399 ± 0.11^c^ | 0.932 ± 0.39^b^ | 19832.15 ± 4.98^c^ |
| AG + Phytol | 1.462 ± 0.15^a^ | 23.459 ± 0.12^d^ | 1.039 ± 0.51^c^ | 20160.05 ± 5.63^d^ |
| AG + Benzoic acid | 1.921 ± 0.34^d^ | 23.591 ± 0.15^e^ | 1.098 ± 0.51^a^ | 20512.63 ± 6.61^a^ |
| AG + Apigenin | 1.497 ± 0.16^e^ | 23.491 ± 0.07^b^ | 0.956 ± 0.43^b^ | 19751.53 ± 3.93^e^ |
|  |  |  |  |  |
| DPP-IV | 1.418 ± 0.19^a^ | 26.985 ± 0.12^a^ | 1.211 ± 0.77^a^ | 25134.84 ± 451.90^a^ |
| DPP-IV + Diprotin A | 1.756 ± 0.23^b^ | 27.118 ± 0.11^b^ | 1.252 ± 0.78^a^ | 24891.80 ± 378.62^b^ |
| DPP-IV + Luteolin | 2.093 ± 0.29^c^ | 27.101 ± 0.09^b^ | 1.283 ± 0.76^c^ | 24757.82 ± 359.15^b^ |
| DPP-IV + Xylocaine | 1.891 ± 0.17^d^ | 27.327 ± 0.13^c^ | 1.164 ± 0.58^b^ | 24637.47 ± 373.40^b^ |
| DPP-IV + Apigenin | 1.905 ± 0.28^d^ | 27.297 ± 0.10^c^ | 1.182 ± 0.58^b^ | 25601.66 ± 395.67^c^ |
| DPPIV + Pinocembrin | 1.982 ± 0.180^d^ | 27.327 ± 0.11^c^ | 1.257 ± 0.77^a^ | 25185.67 ± 488.93^d^ |
| DPPIV + Cistanoside D | 1.787 ± 0.17^b^ | 27.133 ± 0.09^b^ | 1.187 ± 0.58^b^ | 25157.60 ± 474.17^a^ |
|  |  |  |  |  |
| PTP-1B | 1.681 ± 0.24^a^ | 18.921 ± 0.07^a^ | 1.277 ± 0.87^a^ | 13194.41 ± 312.84^a^ |
| PTP-1B + Ursolic acid | 1.498 ± 0.16^b^ | 18.864 ± 0.07^b^ | 1.109 ± 0.57^b^ | 12749.91 ± 332.30^b^ |
| PTP-1B + Chlorogenic acid | 1.242 ± 0.11^c^ | 18.844 ± 0.05^b^ | 0.987 ± 0.48^c^ | 12804.77 ± 267.42^c^ |
| PTP-1B + Naringenin | 1.291 ± 0.12^c^ | 18.911 ± 0.10^a^ | 1.076 ± 0.48^b^ | 12714.82 ± 400.36^b^ |
| PTP-1B + Pinocembrin | 1.427 ± 0.21^b^ | 18.869 ± 0.07^b^ | 1.182 ± 0.65^c^ | 12560.01 ± 318.85^d^ |
| PTB1B + TZD | 1.162 ± 0.08^d^ | 18.871 ± 0.07^b^ | 0.959 ± 0.47^d^ | 12574.774 ± 321.39^d^ |
| PTB1B + TCA | 1.319 ± 0.15^e^ | 19.043 ± 0.09^c^ | 1.116 ± 0.54^e^ | 13074.964 ± 304.36^e^ |
|  |  |  |  |  |
| AR | 2.126 ± 0.216^a^ | 19.342 ± 0.08^a^ | 1.089 ± 0.52^a^ | 13783.47 ± 357.35^a^ |
| AR + Ranirestat | 2.217 ± 0.30^b^ | 19.357 ± 0.09^a^ | 1.051 ± 0.58^b^ | 13503.46 ± 291.10^b^ |
| AR + Chlorogenic acid | 1.754 ± 0.22^c^ | 19.215 ± 0.07^b^ | 1.022 ± 0.56^c^ | 13132.35 ± 272.52^c^ |
| AR + Naringenin | 1.876 ± 0.27^d^ | 19.327 ± 0.07^a^ | 1.070 ± 0.67^a^ | 13343.60 ± 277.74^d^ |
| AR + Luteolin | 1.491 ± 0.18^e^ | 19.165 ± 0.09^c^ | 1.024 ± 0.63^c^ | 13116.07 ± 294.48^e^ |
| AR + Isoflavone | 1.735 ± 0.25^c^ | 19.314 ± 0.09^a^ | 1.045 ± 0.79^b^ | 13316.77 ± 255.46^e^ |

AG: Alpha-glucosidase; AR: Aldose reductase; DPP-IV: dipeptidyl peptidase-IV; PTP-1B: protein tyrosine phosphatase-1B; TZD: 1,2,4,5-tetrazine-3,6-diamine; TCA: trans cinnamic acid; RMSD: Root mean square deviation; RMSF: Root mean square fluctuation; Rg: Radius of gyration; SASA: Solvent accessible surface area

^a,b,c,d,e^Values with different superscript letters down the column for each parameter are significantly different from each other (p<0.05)

Table 4. Identified interactions between the top phytoconstituents and carbohydrate enzymes’ amino acid residues

| **Phytocompounds** | **Number of interactions** | **Number of H-bonds and interaction residues** | **Number of van der Waal forces and interaction residues** | **Other important interactions and residues** |
| --- | --- | --- | --- | --- |
| AG-Acarbose | 30 | 4 (Asp202, Arg200, Gln170, Asn301) | 20 (Phe297, Gly228, Tyr389, Phe397, Glu231, Met302, Leu300, Val335, Val334, Leu227, His105, Phe206, Ile146, Phe166, Asp62, Tyr65, Arg404, His332, Phe147, Thr203) | 6 (Arg400, Glu271, Asp333, Pro230, Ala229, Arg340) |
| AG-Apigenin | 16 | 2 (Phe294, Asp330) | 9 (Glu228, Phe394, Leu390, Arg397, Val331, Leu297, Asn298, Asp295, Met299) | 5 (Leu224, Ala226, Tyr386, Pro227, Gly225 |
| AG-Benzoic acid | 15 | 3 (Asn328, Gly225, Glu268) | 10 (Met299, Asn298, Val331, Leu297, Gly270, Ile143, Phe163, Asp330, Arg197, Phe203) | 2 (Hie329, Phe294) |
| AG-Isoflavone | 16 | - | 14 (Ile143, Ala226, Gly225, Tyr232, Phe203, Leu241, Gly270, Asn202, Ile269, Glu268, Thy200, Phe294, Asp330, Arg397) | 2 (Phe163, Val331) |
| AG-Phytol | 23 | 1 (Asp199) | 17 (Leu224, Gly225, Asn328, Glu268, Arg197, Hie102, Tyr200, Val89, Phe163, Leu164, Asp330, Trp48, Phe144, Ile143, Asn298, Met299, Arg337) | 5 (Tyr62, Leu297, Tyr292, Phe294, Val331) |
| DPP-IV-Diprotin A | 11 | 2 (Ser593, Tyr510) | 3 (Tyr625, Asn673, Arg88) | 6 (Tyr629, Glu169, Glu168, Asp626, Hid703, Trp592) |
| DPP-IV-Xylocaine | 15 | 4 (Gln516, Arg392, Tyr510, Lys517) | 8 (Tyr926, Cys514, Phe320, Tyr548, Glu168, Asn673, Hid703, Ser593) | 3 (Asp672, Arg88, Ser515) |
| DPP-IV-Pinocembrin | 12 | 3 (Trp592, Val509, Gly591) | 8 (Gly595, Gly596, Ser593, Tyr594, Tyr629, Trp590, Asp508, Lys517) | 1 (Tyr510) |
| DPP-IV-Cistanoside D | 11 | 1 (Tyr11) | 6 (Tyr715, Hid711, Trp592, Ser172, Tyr510, Tyr594) | 4 (Ser593, Ala706, Gly704, Hid703) |
| DPP-IV-Apigenin | 15 | 3 (Asp508, Tyr625, Ser593) | 11 (Val509, Gly591, Lys517, Trp590, Asn525, Tyr715, Hid703, Tyr510, Val674, Asn673, Gly704) | 1 (Trp592) |
| PTP-1B-Ursolic acid | 13 | - | 7 (Asp44, Met254, Gly255, Gln258, Ile215, Ser212, Leu115) | 6 (Val45, Tyr42, Lys112, Lys116, Arg41, Ala213) |
| AR-Naringenin | 16 | 3 (Asp43, Trp20, Gly18) | 9 (Tyr48, Gln183, Thr19, Ser263, Pro261, Ser210, Leu212, Pro211, Ser214) | 4 (Lys77, Ile260, Tyr209, Lys262, |
| PTP-1B-TCA | 13 | 2 (Phe3, Glu2) | 8 (Glu4, Asp241, Ile242, Val240, Cys227, Pro237, Ala274, Ile271) | 3 (Lys1, Met231, Val270) |
| PTP-1B-TZD | 9 | 3 (Thr134, Asn158, Asn135) | 4 (Glu97, Glu93, Trp96, Thr160) | 2 (Hie56, Leu136) |
| PTP-1B-Pinocembrin | 9 | 2 (Ala213, Gln258) | 5 (Arg41, Ser212, Gly255, Met254, Val45) | 2 (Tyr42, Ile215) |
| PTP-1B-Naringenin | 10 | 3 (Lys54, Hie56, Lys69) | 4 (Glu97, Ile78, Pro206, Arg52) | 3 (Ile53, Gln98, Leu67) |
| PTP-1B-Chlorogenic acid | 6 | 2 (Ala260, Thr259) | 3 (Asp261, Gln258, Tyr16) | 1 (Ala13) |
| AR-Ranirestat | 11 | 2 (Try20, Ala299) | 6 (Tyr209, Trp111, Lys21, Trp79, Phe122, Phe121) | 3 (Val297, Leu300, Cys298) |
| AR-Luteolin | 25 | 7 (Asp216, Glu185, Trp111, Cys298, Lys77, Asp43, Ser210) | 14 (Leu212, Pro211, Pro261, Pro215, Ser214, Val297, Arg296, Phe311, Ala299, Asn160, Hie110, Tyr48, Gln183, Ile260) | 4 (Trp20, Lys262, Gly18, Tyr209) |
| AR-Isoflavone | 4 | - | 4 (Thr286, Ser290, Leu289, Asn292) |  |
| AR-Chlorogenic acid | 20 | 7 (Asp43, Thr19, Trp20, Ser210, Pro218, Asp216, Gly18) | 11 (Ile260, Gln183, Lys77, Tyr48, Hie110, Trp111, Pro215, Ser214, Arg217, Tyr209, Lys262) | 2 (Cys298, Val297) |
| AR-Narengenin | 16 | 2 (Asp43, Trp20) | 9 (Gln183, Tyr48, Thr19, Ser263, Pro261, Ser210, Leu212, Pro211, Ser214) | 5 (Lys77, Gly18, Lys262, Tyr209, Ile260) |

AG: Alpha-glucosidase; AR: Aldose reductase; DPP-IV: Dipeptidyl peptidase; PTP-1B: Protein tyrosine phosphatase; TZD: 1,2,4,5-tetrazine-3,6-diamine; TCA: trans cinnamic acid;

Table 5. ADME properties prediction for phytocompounds and reference standards

| **Compounds** | **Properties** | | | | | | | | | | | |
| --- | --- | --- | --- | --- | --- | --- | --- | --- | --- | --- | --- | --- |
|  | **Bioavailability score** | **Water solubility** | **Lipophilicity (ilogP)** | **GIT absorption** | **Hydrogen bond acceptors** | **Hydrogen bond donors** | **Lipinski rule** | **Cytochrome inhibitors** | | | | |
|  |  |  |  |  |  |  |  | **CYP1A2** | **CYP2C19** | **CYP2C9** | **CYP2D6** | **CYP3A4** |
| Acarbose | 0.17 | Highly soluble | 0.63 | Low | 19 | 14 | N | N | N | N | N | N |
| Diprotin A | 0.55 | Very soluble | 2.14 | High | 5 | 3 | Y | N | N | N | N | N |
| Ursolic acid | Poorly soluble |  | 3.95 | Low | 3 | 2 | Y | N | N | N | N | N |
| Ranirestat | 0.55 | Soluble | 2.15 | High | 5 | 1 | Y | N | N | N | N | N |
| Apigenin | 0.55 | Moderate | 1.89 | High | 5 | 3 | Y | Y | N | N | Y | Y |
| Benzoic acid | 0.85 | Soluble | 1.11 | High | 2 | 1 | Y | N | N | N | N | N |
| Catalposide | 0.17 | Soluble | 1.65 | Low | 12 | 6 | N | N | N | N | N | N |
| Chlorogenic acid | 0.11 | Soluble | 0.96 | Low | 9 | 6 | Y | N | N | N | N | N |
| Cistanoside D | 0.17 | Soluble | 2.59 | Low | 15 | 7 | N | N | N | N | N | N |
| Glucopyranoside | 0.55 | Highly soluble | 0.24 | Low | 6 | 5 | Y | N | N | N | N | N |
| Isoflavone | 0.55 | Soluble | 2.51 | High | 2 | 0 | Y | Y | Y | N | N | N |
| Luteolin | 0.55 | Soluble | 1.86 | High | 6 | 4 | Y | Y | N | N | Y | Y |
| Naringenin | 0.55 | Soluble | 1.75 | High | 5 | 3 | Y | Y | N | N | N | Y |
| Phytol | 0.55 | Moderately soluble | 4.71 | Low | 1 | 1 | Y | N | N | Y | N | N |
| Pinocembrin | 0.55 | soluble | 2.11 | High | 4 | 2 | Y | Y | Y | N | N | N |
| 1,2,4,5- tetrazine- 3,6 diamine | 0.55 | Very soluble | 1.56 | High | 4 | 2 | Y | N | N | N | N | N |
| Trans-cinnamic acid | 0.85 | Soluble | 1.55 | High | 2 | 1 | Y | N | N | N | N | N |
| Xylocaine | 0.55 | Soluble | 2.86 | High | 2 | 1 | Y | N | N | N | Y | N |

CYP: Cytochrome; Y: Yes; N: No

b

a








d

c

**Fig. 1** Root Mean Square Deviation (RMSD) plots of comparison between the studied enzymes [**a** (alpha-glucosidase), **b** (Dipeptidyl peptidase-IV), **c** (Protein tyrosine phosphatase-1B) and **d** (aldose reductase) *C. cujete* compounds and standard molecules (acarbose, Diprotin A, ursolic acid and ranirestat)] determined over 100 ns molecular dynamics simulations. DPP-IV; dipeptidyl peptidase-IV; PTP-1B; protein tyrosine phosphatase-1B

CODES: 3W37- alpha-glucosidase; 1IEI- aldose reductase

b

a








d








c

**Fig. 2** Radius of Gyration (Rg) plots of comparison between the studied enzymes [**a** (alpha-glucosidase), **b** (Dipeptidyl peptidase-IV), **c** (Protein tyrosine phosphatase-1B) and **d** (aldose reductase) and *C. cujete* compounds and standard molecules (acarbose, Diprotin A, ursolic acid and ranirestat)] determined over 100 ns molecular dynamics simulations. DPP-IV; dipeptidyl peptidase-IV; PTP-1B; protein tyrosine phosphatase 1B

CODES: 3W37- alpha-glucosidase; 1IEI- aldose reductase

b

a




d

c

**Fig. 3** Root Mean Square Fluctuation (RMSF) plots of comparison between the studied enzymes [**a** (alpha-glucosidase), **b** (Dipeptidyl peptidase-IV), **c** (Protein tyrosine phosphatase-1B) and **d**(aldose reductase)] *C. cujete* compounds and standard molecules (acarbose, Diprotin A, ursolic acid and ranirestat)] determined over 100 ns molecular dynamics simulations. DPP-IV; dipeptidyl peptidase-IV; PTP-1B; protein tyrosine phosphatase 1B

CODES: 3W37- alpha-glucosidase; 1IEI- aldose reductase

b

a




d

c

**Fig. 4** Solvent Accessible Surface Area (SASA) plots of comparison between the studied enzymes [**a** (alpha-glucosidase), **b** (Dipeptidyl peptidase-IV), **c** (Protein tyrosine phosphatase-1B and **d** (aldose reductase)] *C. cujete* compounds and standard molecules (acarbose, Diprotin A, ursolic acid and ranirestat)] determined over 100 ns molecular dynamics simulations. DPP-IV; dipeptidyl peptidase-IV; PTP-1B; protein tyrosine phosphatase 1B

CODES: 3W37- alpha-glucosidase; 1IEI- aldose reductase


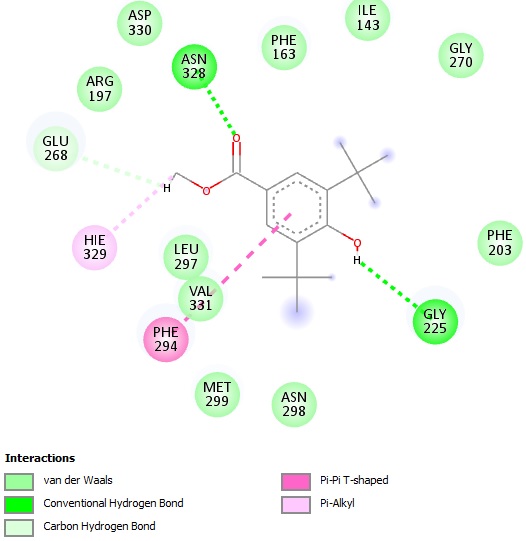


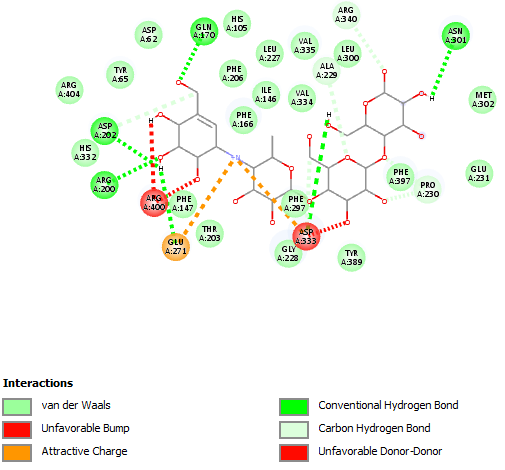


a

b

d


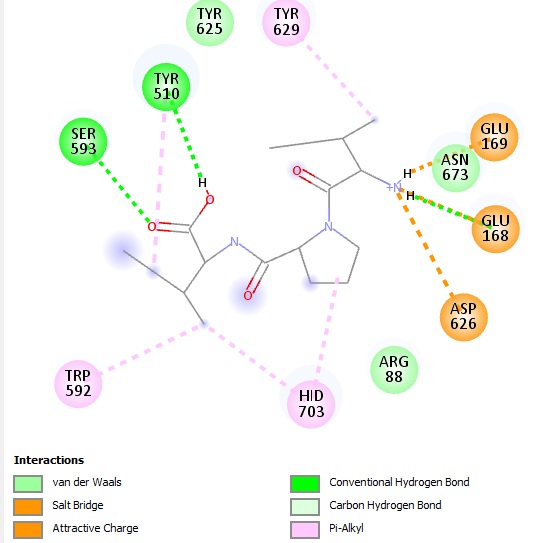


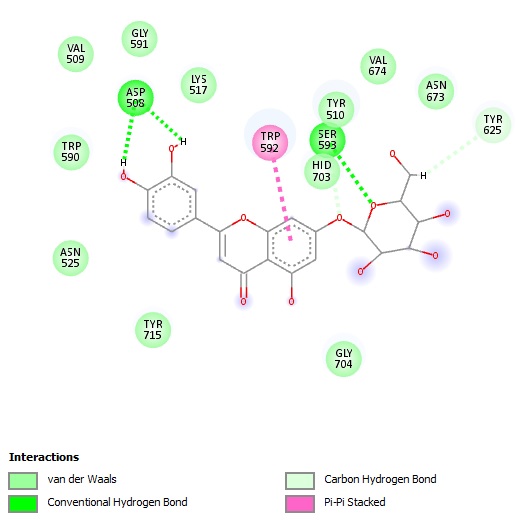


c

f

e


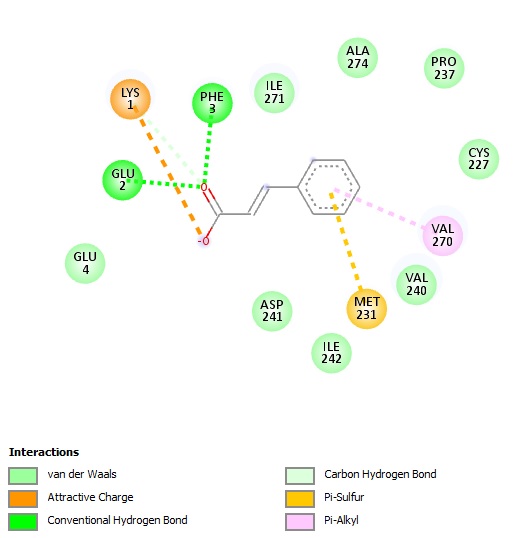


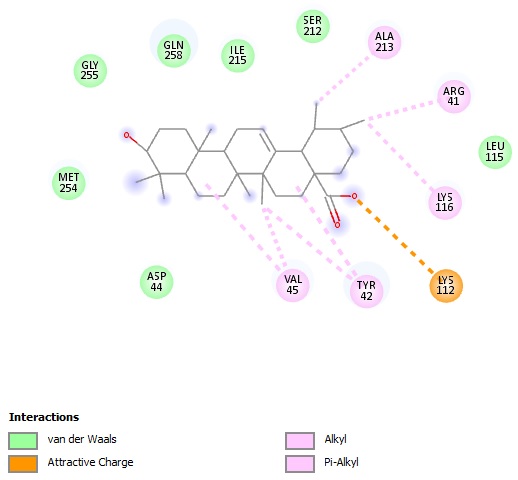


h

g


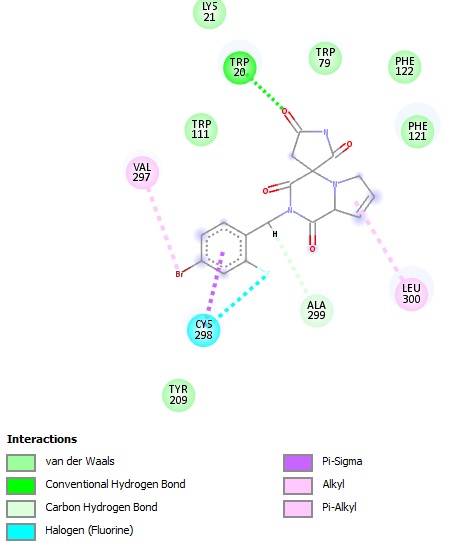


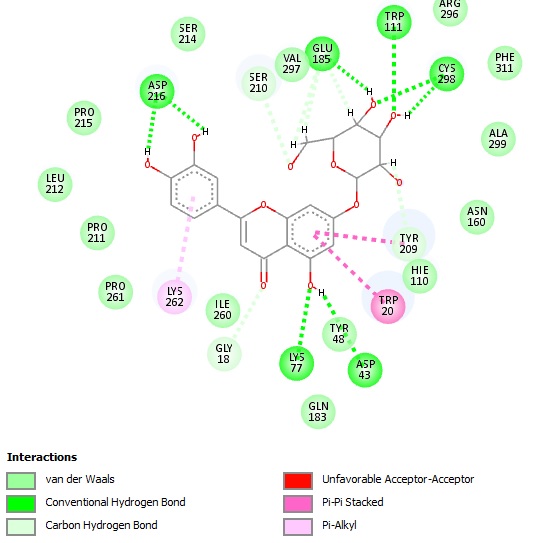


**Fig. 5** Plots of interaction of (**a**) alpha-glucosidase-acarbose, (**b**) alpha-glucosidase-benzoic acid, (**c**) DPP-IV-luteolin, (**d**) DPP-IV-Diprotin A (**e**) PTP-1B-ursolic acid, (**f**) PTP-1B-trans cinnamic acid, (**g**) aldose reductase-luteolin, (**h**) aldose reductase-ranirestat

**Supplementary Materials**

Table S1: Docking scores (kcal/mol) of *C. cujete* phytochemicals against type 2 diabetes mellitus drug targets

| **Compounds** | **Alpha-Glucosidase PDB:3W37** | **DPP-IV PDB:1WCY** | **Protein Tyrosine Phosphatase 1B (2HNQ)** | **Aldose Reductase PDB: 1IEI** |
| --- | --- | --- | --- | --- |
| 1,2.4,5-Tetrazine-3,6-diamine | -5.0 | -4.6 | -5.9 | -5.2 |
| 1,2-ethenediamine | -4.9 | -2.4 | -2.9 | -3.0 |
| 1,3,5-Triazine-2,4,6-triamine | -5.5 | -3.0 | -5.6 | -5.9 |
| 1-Tridecanal | -4.8 | -2.7 | -3.7 | -6.0 |
| 2,3-dihydro-3,5—dihydroxy-6-methyl-4H-pyran-4-one | -5.9 | -4.0 | -5.4 | -5.8 |
| 2,5-difluorophenylhydrazine | -4.6 | -3.9 | -4.8 | -6.8 |
| 2,5-dimethyloxazolidine | -4.8 | -3.8 | -3.8 | -4.4 |
| 2-Galactopyranose | -3.4 | -3.2 | -3.1 | -4.8 |
| 2-tricedene-11-ynedial | -2.6 | -3.2 | -4.3 | -6.8 |
| 3,5-dimethyl-1H-pyrazole | -5.7 | -4.9 | -5.0 | -5.5 |
| 3-pentanone | -5.4 | -3.6 | -4.0 | -4.2 |
| 4H-pyran-4-one | -3.2 | -3.9 | -4.0 | -4.7 |
| 4-mercaptophenol | -4.0 | -4.3 | -4.2 | -5.5 |
| 5-hydroxymethylfurfural | -5.4 | -4.0 | -4.8 | -6.0 |
| 9,17-octadecadienal | -5.3 | -4.0 | -3.6 | -7.0 |
| Acrylic acid | -4.2 | -3.8 | -4.1 | -4.3 |
| Apigenin | -7.3 | -5.7 | -5.5 | -8.4 |
| Aureonitol | -7.1 | -4.6 | -4.6 | -7.7 |
| Benzene propanoic acid | -6.9 | -4.0 | -5.6 | -7.4 |
| Benzoic acid | -7.2 | -5.1 | -2.7 | -7.6 |
| Butyl-2-nitropropanoate | -3.7 | -5.3 | -5.0 | -5.8 |
| Catalpol | -5.2 | -4.7 | -3.5 | -6.5 |
| Catalposide | -3.6 | -5.5 | -2.7 | -8.4 |
| Chlorogenic acid | -4.5 | -4.5 | -6.2 | -9.4 |
| Cistanoside C | -4.7 | -5.3 | -3.2 | -8.7 |
| Cistanoside D | -5.3 | -5.7 | -5.4 | -8.0 |
| D-Galactopyranose | -3.4 | -4.1 | -5.3 | -5.9 |
| Funarion | -4.2 | -4.0 | -3.4 | -5.7 |
| Furaneol | -4.0 | -4.0 | -4.7 | -5.7 |
| Furfural | -3.7 | -3.9 | -4.4 | -4.7 |
| Helanill | -5.5 | -2.5 | -4.0 | -6.6 |
| Helenalin | -4.9 | -5.2 | -4.9 | -7.6 |
| Hexadecanol | -5.3 | -3.2 | -2.7 | -6.5 |
| Hexanoic acid | -3.9 | -4.9 | -4.5 | -5.3 |
| Hydroxycinnamic acid | -4.2 | -4.1 | -5.5 | -7.1 |
| Indolizine | -5.6 | -4.1 | -4.6 | -5.9 |
| Isochavacine | -7.1 | -5.0 | -2.9 | -8.5 |
| Isoflavone | -7.7 | -4.9 | -5.2 | -9.6 |
| Isopropyl myristate | -4.2 | -5.3 | -2.9 | -7.0 |
| kaur-16-ene | -4.8 | -5.5 | -1.2 | -8.3 |
| Luteolin | -5.7 | -6.7 | -1.9 | -9.7 |
| Naringenin | -7.0 | -5.6 | -5.7 | -9.9 |
| Neophytadiene | -7.1 | -3.9 | -3.1 | -7.4 |
| Palmitic acid | -4.0 | -4.8 | -4.0 | -6.9 |
| Phenol | -5.1 | -4.0 | -5.5 | -7.2 |
| Phosphonous acid | -4.3 | -3.5 | -3.9 | -8.7 |
| p-hydroxybenzoic acid | -5.3 | -3.8 | -5.0 | -6.9 |
| Phytol | -7.4 | -4.6 | -3.2 | -8.6 |
| Pinocembrin | -4.9 | -5.7 | -5.9 | -8.7 |
| Protocatechuic acid | -3.8 | -4.2 | -4.2 | -6.2 |
| Pyrazole | -5.5 | -3.1 | -3.8 | -3.8 |
| Sulochrin | -4.9 | -5.0 | -4.1 | -7.3 |
| t-butylthiothioacetic acid | -5.2 | -4.5 | -3.8 | -4.3 |
| Trans-cinnamic acid | -5.0 | -4.6 | -5.7 | -7.1 |
| Uracil | -4.8 | -2.9 | -4.5 | -5.4 |
| Xycaine | -4.6 | -5.7 | -3.3 | -8.3 |
|  |  |  |  |  |
| Controls |  |  |  |  |
| Ranirestat |  |  |  | -9.0 |
| Ursolic acid |  |  | -7.4 |  |
| Acarbose | -5.0 |  |  |  |
| Diprotin A |  | -5.5 |  |  |

B

A


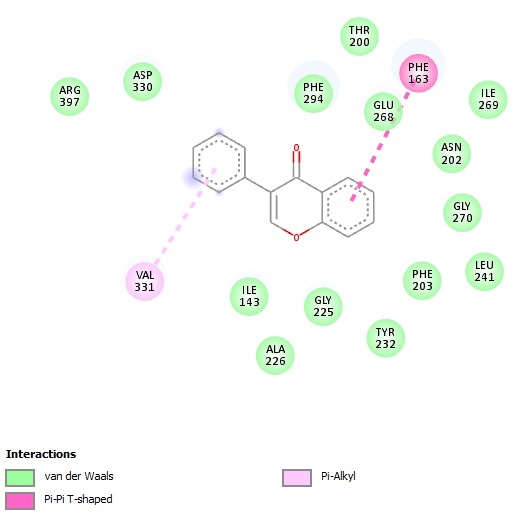


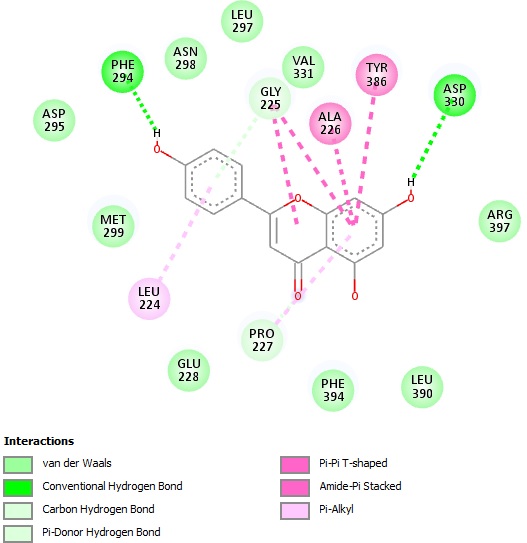


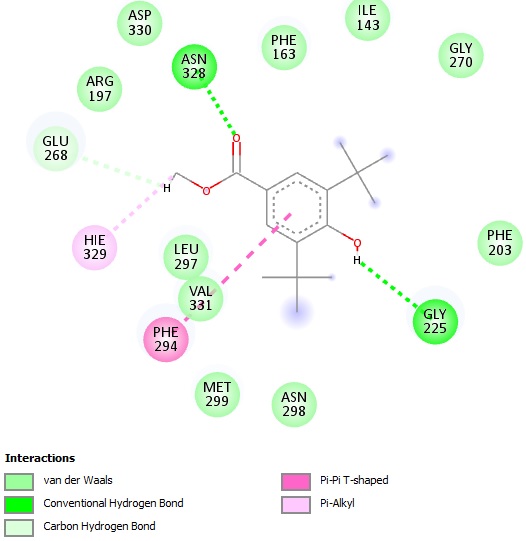


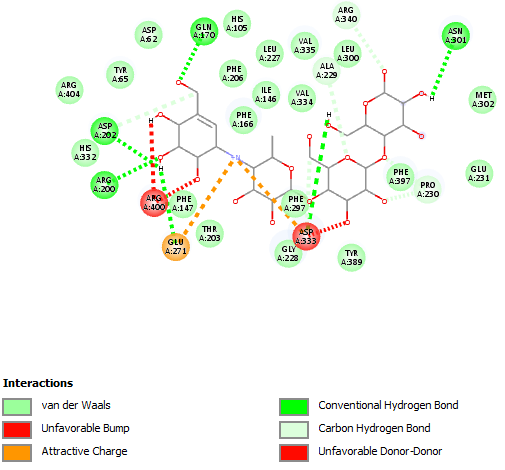


D

C

F

E


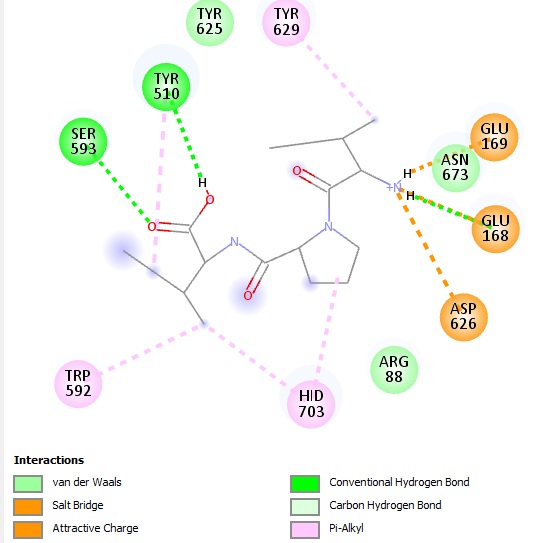


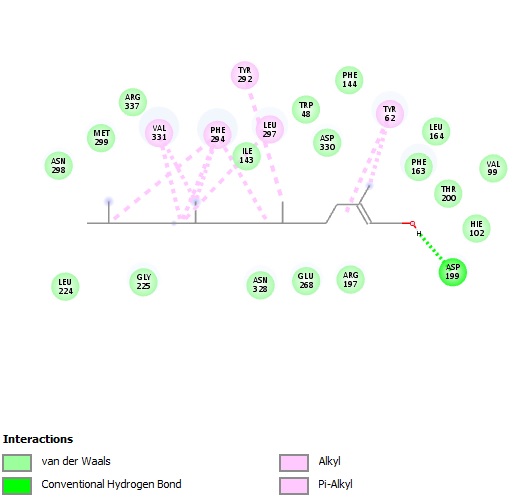


G


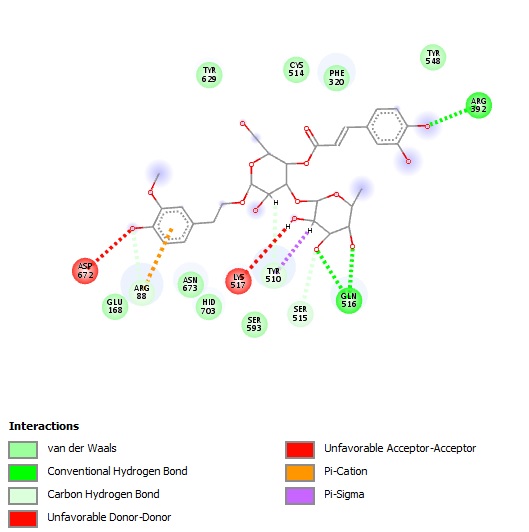


H


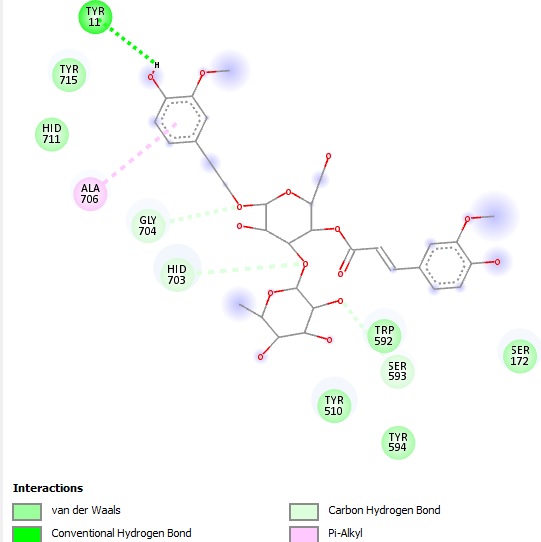


J

I


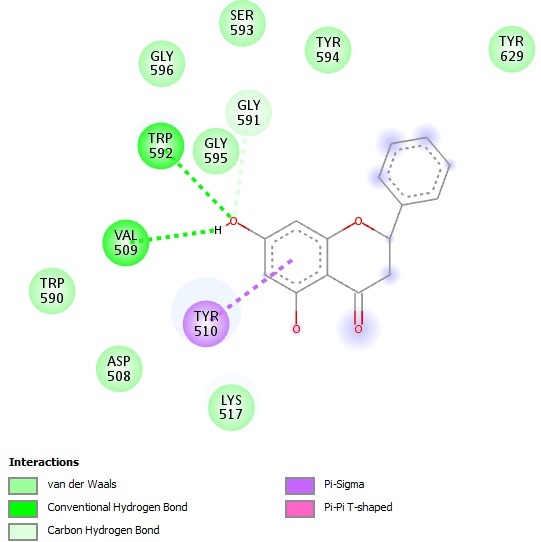


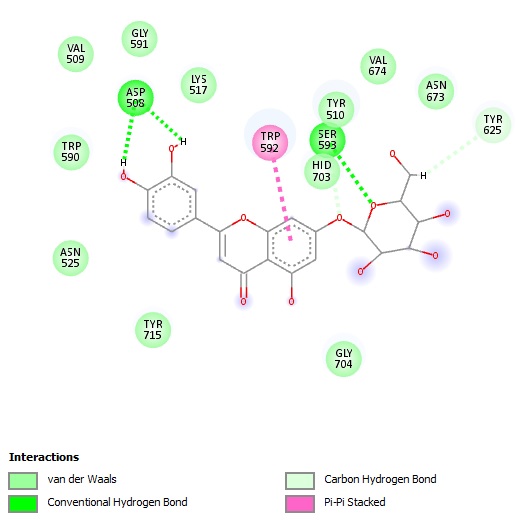


L

K


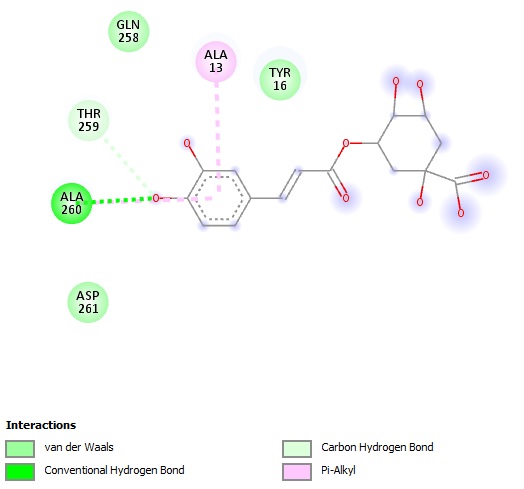


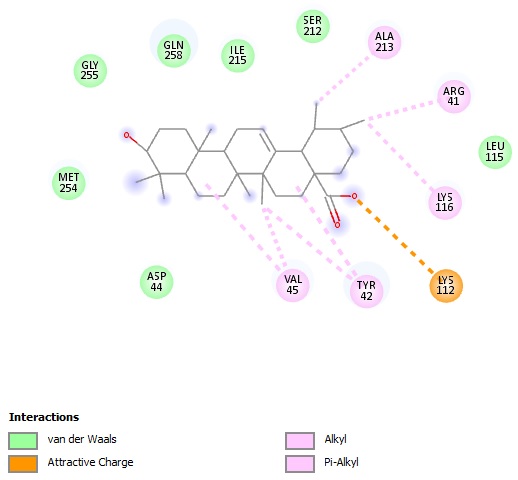


N

M


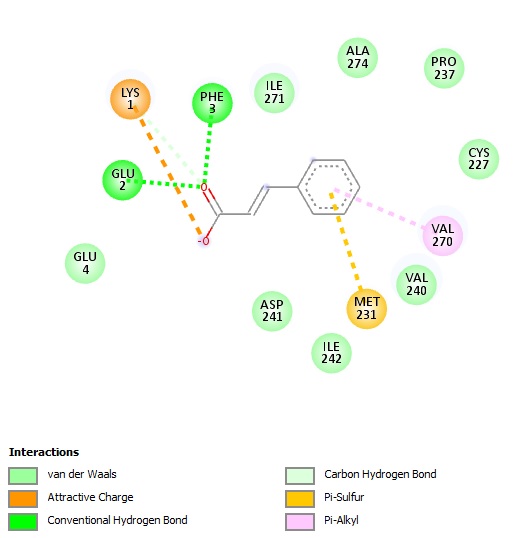


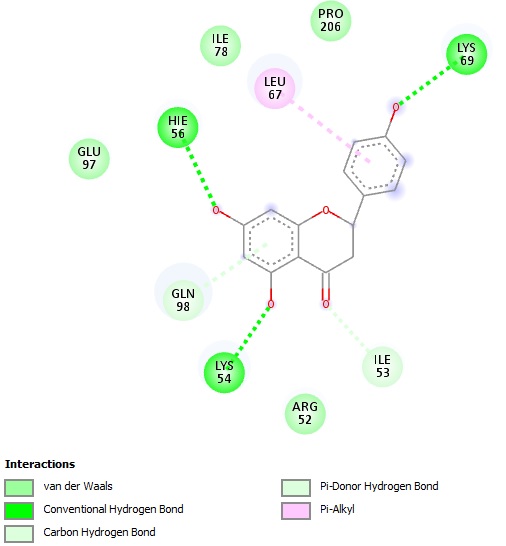


P

O


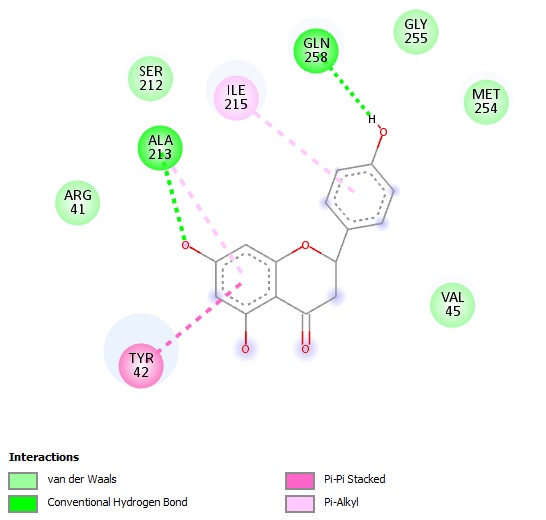


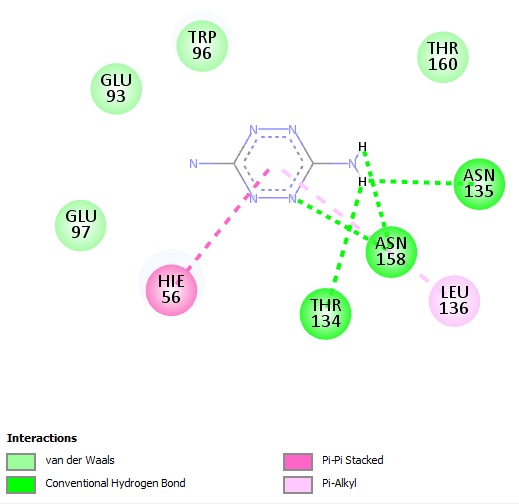


R

Q


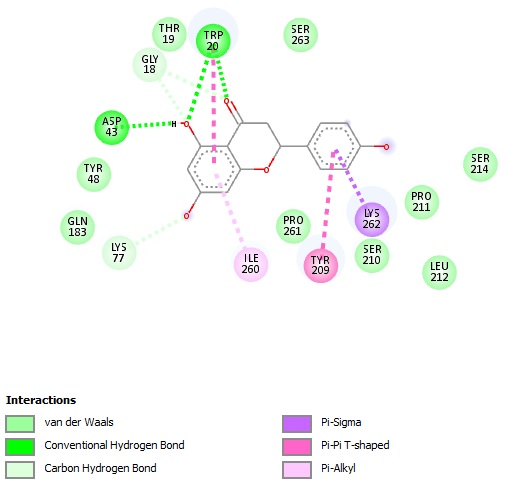


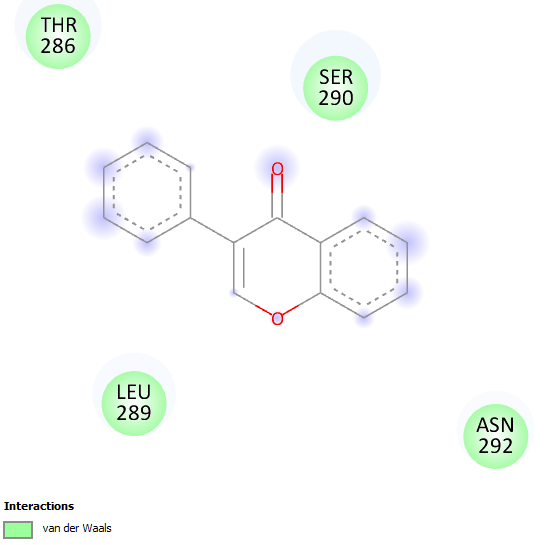


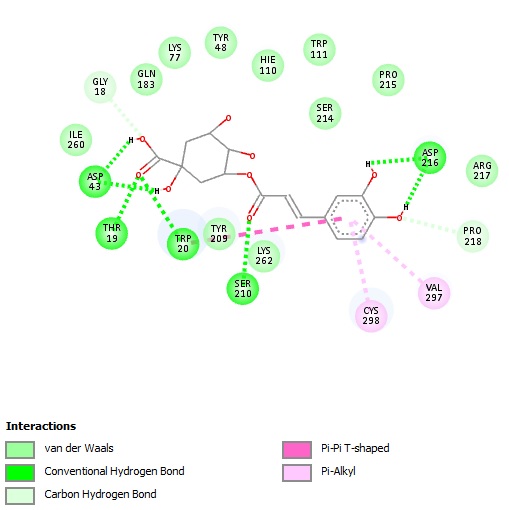


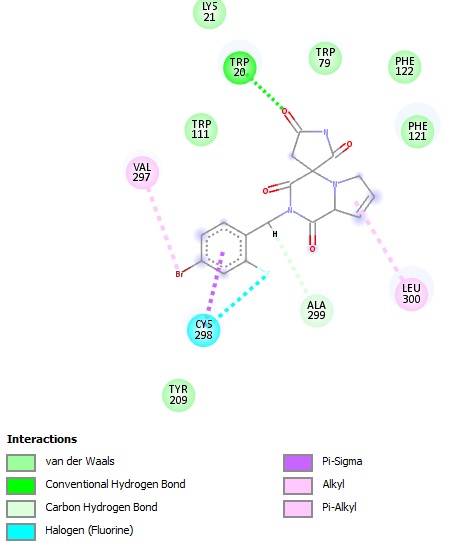


T

S

U


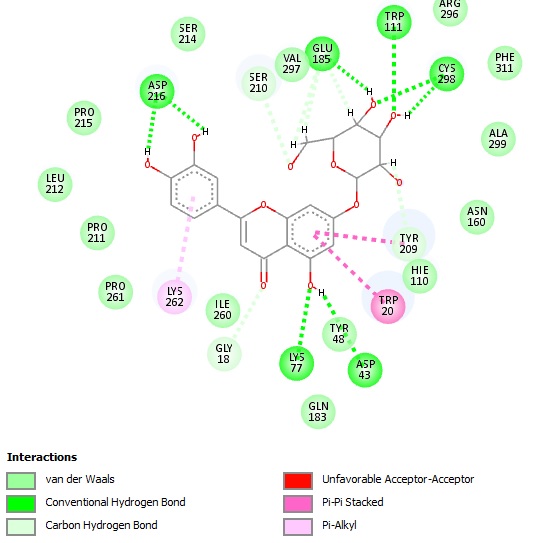


Figure S1. Interaction plots between alpha-glucosidase-acarbose (A), alpha-glucosidase-apigenin (B), alpha-glucosidase-benzoic acid (C), alpha-glucosidase—isoflavone (D), alpha-glucosidase-phytol (E), DPP-IV-Diprotin (F), DPP-IV-xycaine (G), DPP-IV-cistanoside D (H), DPP-IV-pinocembrin (I), DPP-IV-luteolin (J), PTP-1B-ursolic acid (K), PTP-1B-chlorogenic acid (L), PTP-1B-naringenin (M), PTP-1B-trans cinnamic acid (N), PTP-1B-1,2,4,5-tetrazine, 3,6-diamine (O), PTP-1B-pinocembrin (P), aldose reductase-ranirestat (Q), aldose reductase-chlorogenic acid (R), aldose reductase-isoflavone (S), aldose reductase-naringenin (T), aldose reductase-luteolin (U)
