# Supplementary material for: Cheminformatics identification of modulators of key carbohydrate-metabolizing enzymes from C. cujete for type-2 diabetes mellitus intervention
Source: J Diabetes Metab Disord. 2023 Jul 1;22(2):1299–317. doi: 10.1007/s40200-023-01249-7 (PMC10638353; doi:10.1007/s40200-023-01249-7)
Supplement: Supplementary file 2 — Supplementary Material 2 [file 40200_2023_1249_MOESM2_ESM.docx]

**Supplementary Materials**

Table S1: Docking scores (kcal/mol) of *C. cujete* phytochemicals against type 2 diabetes mellitus drug targets

| **Compounds** | **Alpha-Glucosidase PDB:3W37** | **DPP-IV PDB:1WCY** | **Protein Tyrosine Phosphatase 1B (2HNQ)** | **Aldose Reductase PDB: 1IEI** |
| --- | --- | --- | --- | --- |
| 1,2.4,5-Tetrazine-3,6-diamine | -5.0 | -4.6 | -5.9 | -5.2 |
| 1,2-ethenediamine | -4.9 | -2.4 | -2.9 | -3.0 |
| 1,3,5-Triazine-2,4,6-triamine | -5.5 | -3.0 | -5.6 | -5.9 |
| 1-Tridecanal | -4.8 | -2.7 | -3.7 | -6.0 |
| 2,3-dihydro-3,5—dihydroxy-6-methyl-4H-pyran-4-one | -5.9 | -4.0 | -5.4 | -5.8 |
| 2,5-difluorophenylhydrazine | -4.6 | -3.9 | -4.8 | -6.8 |
| 2,5-dimethyloxazolidine | -4.8 | -3.8 | -3.8 | -4.4 |
| 2-Galactopyranose | -3.4 | -3.2 | -3.1 | -4.8 |
| 2-tricedene-11-ynedial | -2.6 | -3.2 | -4.3 | -6.8 |
| 3,5-dimethyl-1H-pyrazole | -5.7 | -4.9 | -5.0 | -5.5 |
| 3-pentanone | -5.4 | -3.6 | -4.0 | -4.2 |
| 4H-pyran-4-one | -3.2 | -3.9 | -4.0 | -4.7 |
| 4-mercaptophenol | -4.0 | -4.3 | -4.2 | -5.5 |
| 5-hydroxymethylfurfural | -5.4 | -4.0 | -4.8 | -6.0 |
| 9,17-octadecadienal | -5.3 | -4.0 | -3.6 | -7.0 |
| Acrylic acid | -4.2 | -3.8 | -4.1 | -4.3 |
| Apigenin | -7.3 | -5.7 | -5.5 | -8.4 |
| Aureonitol | -7.1 | -4.6 | -4.6 | -7.7 |
| Benzene propanoic acid | -6.9 | -4.0 | -5.6 | -7.4 |
| Benzoic acid | -7.2 | -5.1 | -2.7 | -7.6 |
| Butyl-2-nitropropanoate | -3.7 | -5.3 | -5.0 | -5.8 |
| Catalpol | -5.2 | -4.7 | -3.5 | -6.5 |
| Catalposide | -3.6 | -5.5 | -2.7 | -8.4 |
| Chlorogenic acid | -4.5 | -4.5 | -6.2 | -9.4 |
| Cistanoside C | -4.7 | -5.3 | -3.2 | -8.7 |
| Cistanoside D | -5.3 | -5.7 | -5.4 | -8.0 |
| D-Galactopyranose | -3.4 | -4.1 | -5.3 | -5.9 |
| Funarion | -4.2 | -4.0 | -3.4 | -5.7 |
| Furaneol | -4.0 | -4.0 | -4.7 | -5.7 |
| Furfural | -3.7 | -3.9 | -4.4 | -4.7 |
| Helanill | -5.5 | -2.5 | -4.0 | -6.6 |
| Helenalin | -4.9 | -5.2 | -4.9 | -7.6 |
| Hexadecanol | -5.3 | -3.2 | -2.7 | -6.5 |
| Hexanoic acid | -3.9 | -4.9 | -4.5 | -5.3 |
| Hydroxycinnamic acid | -4.2 | -4.1 | -5.5 | -7.1 |
| Indolizine | -5.6 | -4.1 | -4.6 | -5.9 |
| Isochavacine | -7.1 | -5.0 | -2.9 | -8.5 |
| Isoflavone | -7.7 | -4.9 | -5.2 | -9.6 |
| Isopropyl myristate | -4.2 | -5.3 | -2.9 | -7.0 |
| kaur-16-ene | -4.8 | -5.5 | -1.2 | -8.3 |
| Luteolin | -5.7 | -6.7 | -1.9 | -9.7 |
| Naringenin | -7.0 | -5.6 | -5.7 | -9.9 |
| Neophytadiene | -7.1 | -3.9 | -3.1 | -7.4 |
| Palmitic acid | -4.0 | -4.8 | -4.0 | -6.9 |
| Phenol | -5.1 | -4.0 | -5.5 | -7.2 |
| Phosphonous acid | -4.3 | -3.5 | -3.9 | -8.7 |
| p-hydroxybenzoic acid | -5.3 | -3.8 | -5.0 | -6.9 |
| Phytol | -7.4 | -4.6 | -3.2 | -8.6 |
| Pinocembrin | -4.9 | -5.7 | -5.9 | -8.7 |
| Protocatechuic acid | -3.8 | -4.2 | -4.2 | -6.2 |
| Pyrazole | -5.5 | -3.1 | -3.8 | -3.8 |
| Sulochrin | -4.9 | -5.0 | -4.1 | -7.3 |
| t-butylthiothioacetic acid | -5.2 | -4.5 | -3.8 | -4.3 |
| Trans-cinnamic acid | -5.0 | -4.6 | -5.7 | -7.1 |
| Uracil | -4.8 | -2.9 | -4.5 | -5.4 |
| Xycaine | -4.6 | -5.7 | -3.3 | -8.3 |
|  |  |  |  |  |
| Controls |  |  |  |  |
| Ranirestat |  |  |  | -9.0 |
| Ursolic acid |  |  | -7.4 |  |
| Acarbose | -5.0 |  |  |  |
| Diprotin A |  | -5.5 |  |  |

B

A


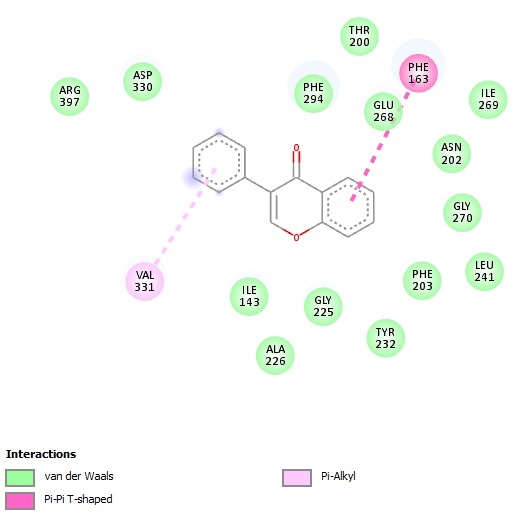


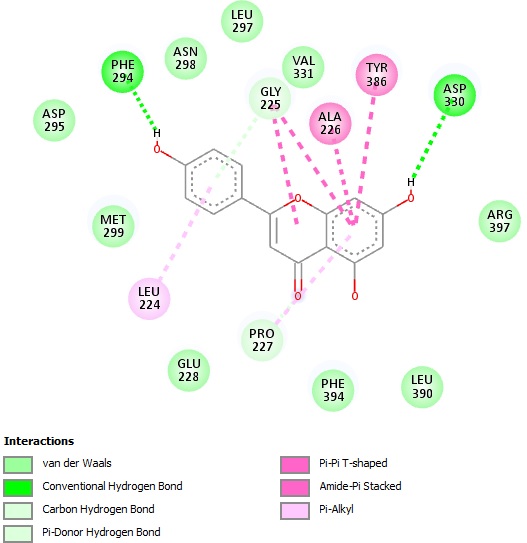


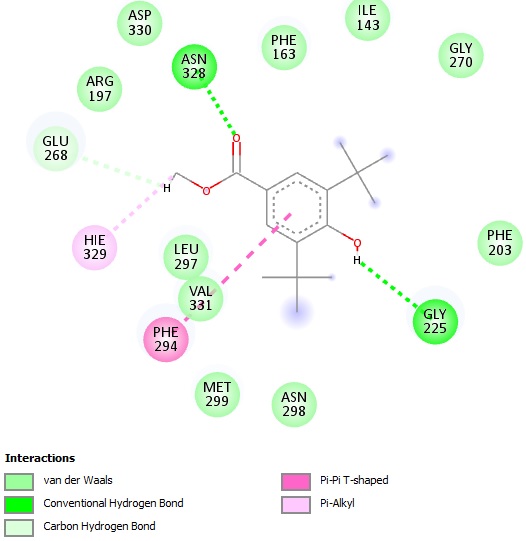


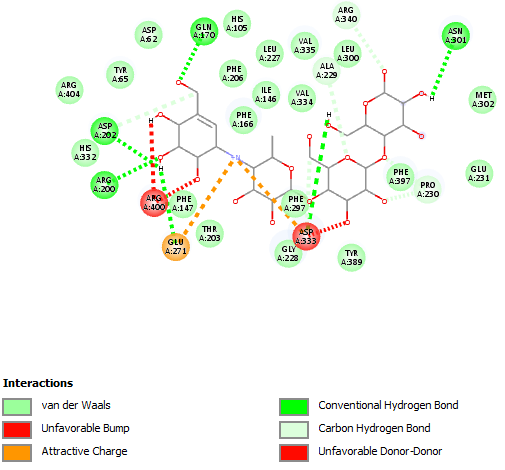


D

C

F

E


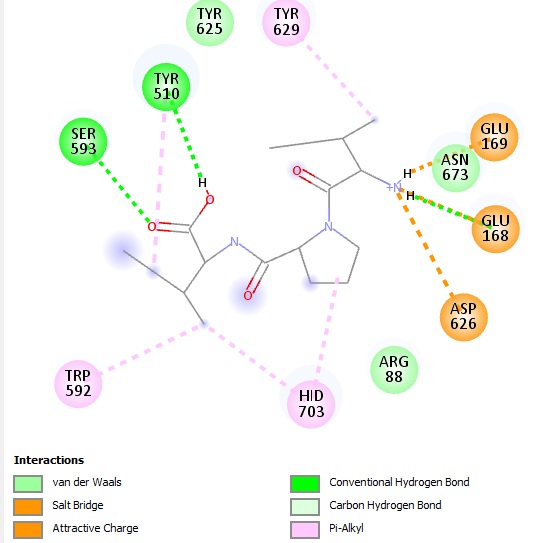


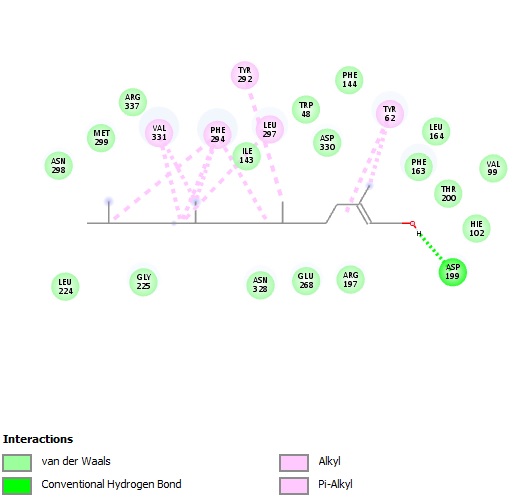


G


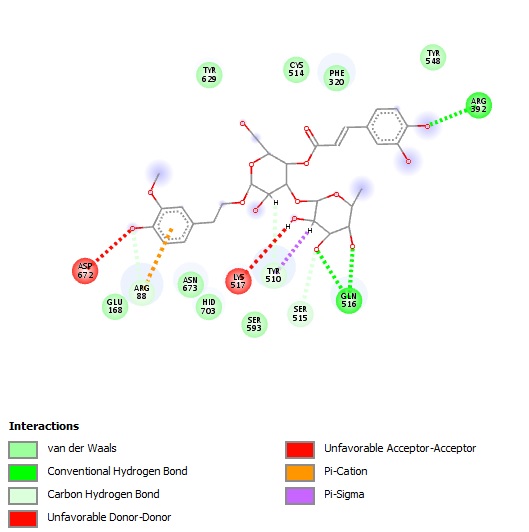


H


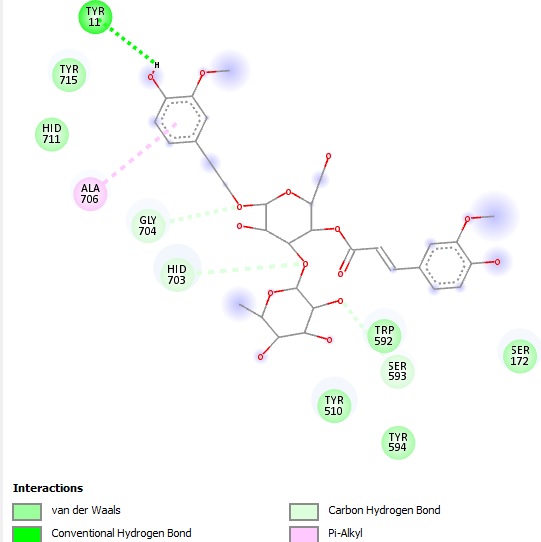


J

I


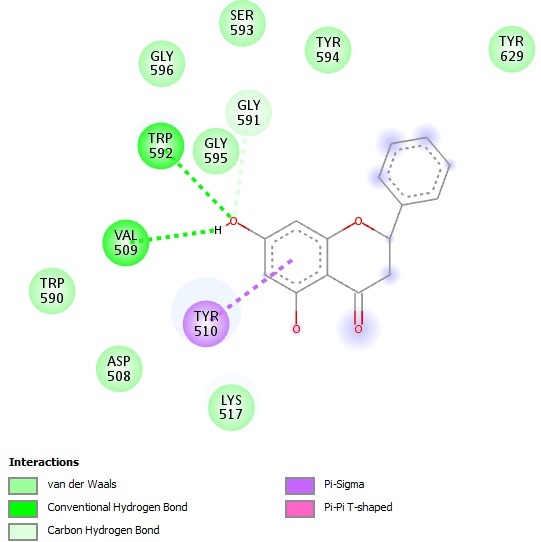


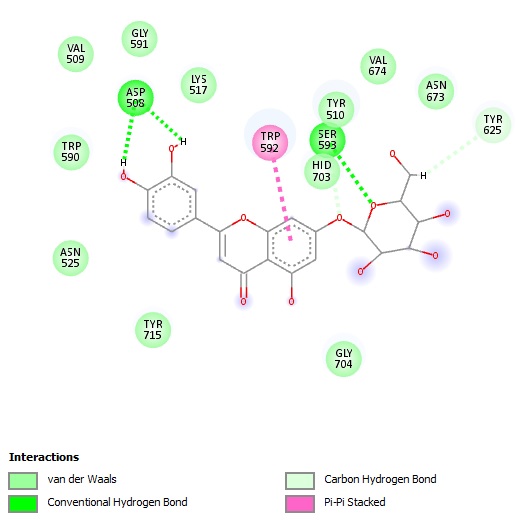


L

K


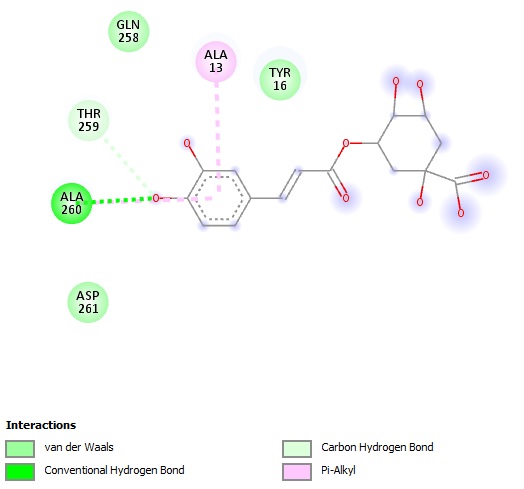


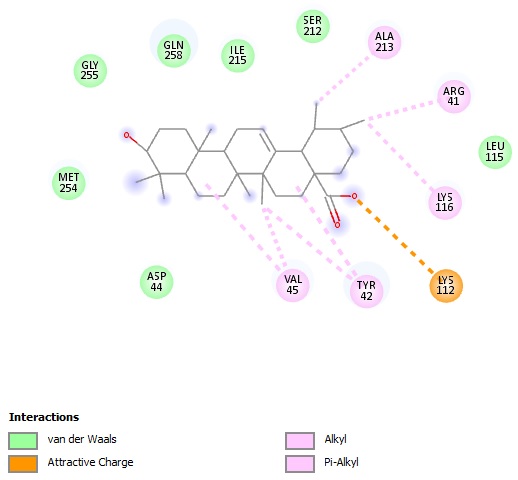


N

M


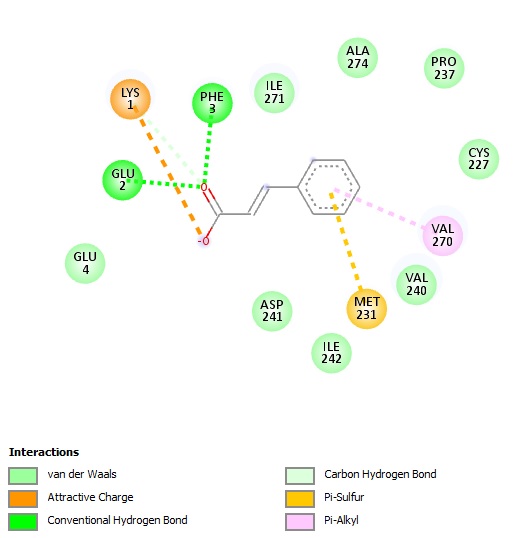


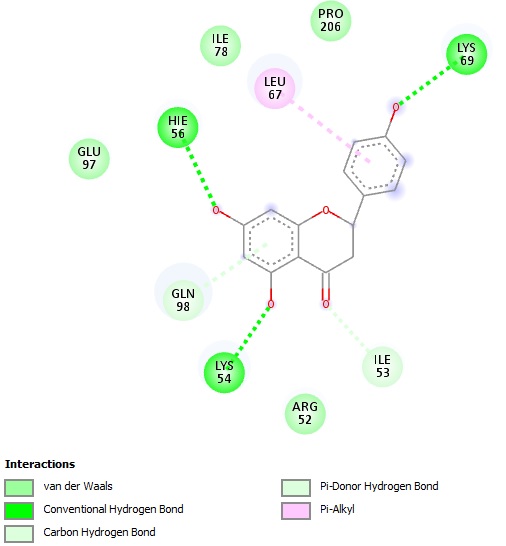


P

O


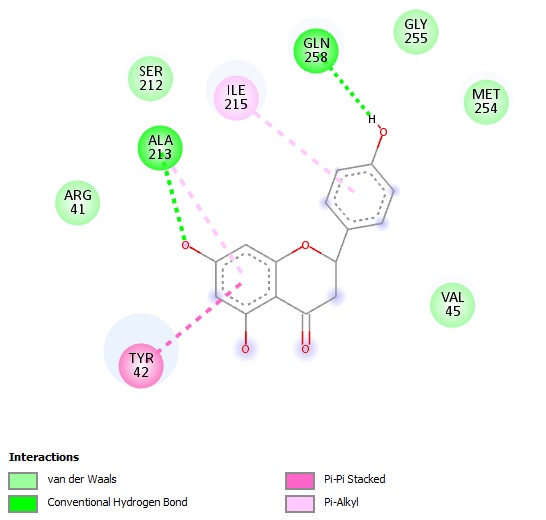


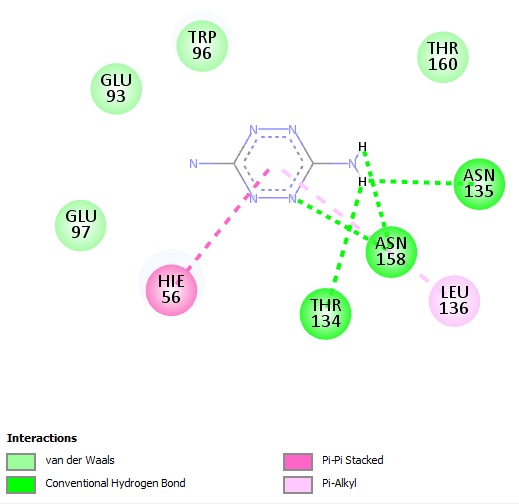


R

Q


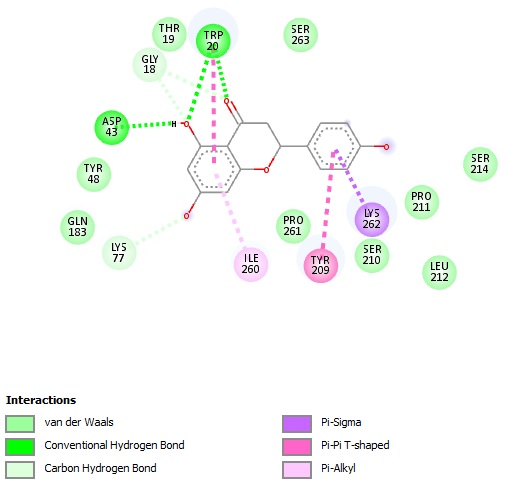


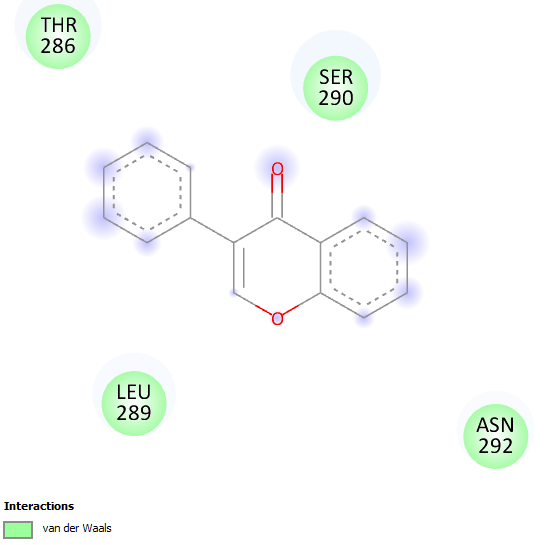


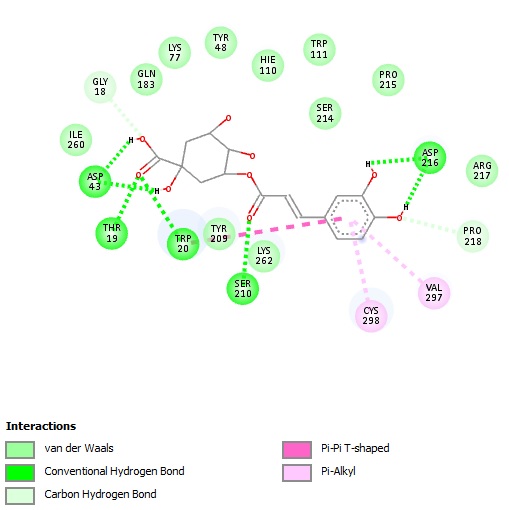


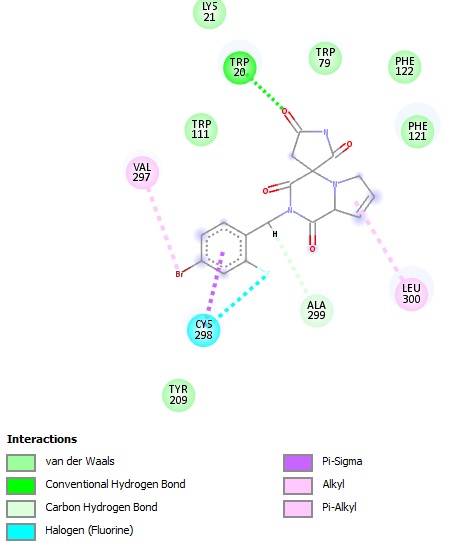


T

S

U


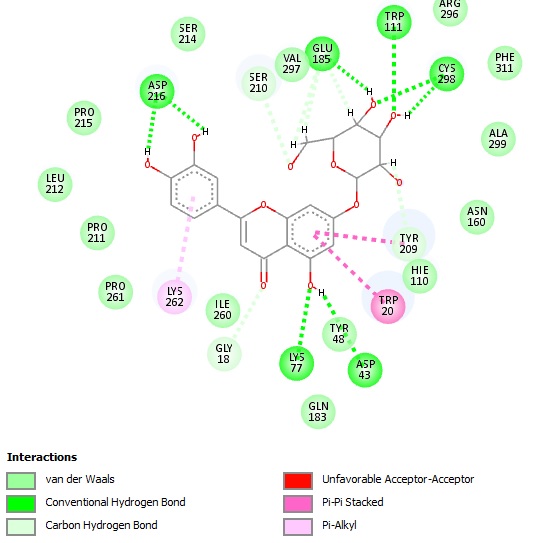


Figure S1. Interaction plots between alpha-glucosidase-acarbose (A), alpha-glucosidase-apigenin (B), alpha-glucosidase-benzoic acid (C), alpha-glucosidase—isoflavone (D), alpha-glucosidase-phytol (E), DPP-IV-Diprotin (F), DPP-IV-xycaine (G), DPP-IV-cistanoside D (H), DPP-IV-pinocembrin (I), DPP-IV-luteolin (J), PTP-1B-ursolic acid (K), PTP-1B-chlorogenic acid (L), PTP-1B-naringenin (M), PTP-1B-trans cinnamic acid (N), PTP-1B-1,2,4,5-tetrazine, 3,6-diamine (O), PTP-1B-pinocembrin (P), aldose reductase-ranirestat (Q), aldose reductase-chlorogenic acid (R), aldose reductase-isoflavone (S), aldose reductase-naringenin (T), aldose reductase-luteolin (U)
